# Interactions between EGFR and EphA2 promote tumorigenesis through the action of Ephexin1

**DOI:** 10.1038/s41419-022-04984-6

**Published:** 2022-06-06

**Authors:** Jeeho Kim, In-Youb Chang, Ho Jin You

**Affiliations:** 1 Laboratory of Genomic Instability and Cancer therapeutics, Chosun University School of medicine, 375 Seosuk-dong, Gwangju, 501-759 South Korea; 2 Department of Pharmacology, Chosun University School of medicine, 375 Seosuk-dong, Gwangju, 501-759 South Korea; 3 Department of Anatomy, Chosun University School of medicine, 375 Seosuk-dong, Gwangju, 501-759 South Korea

**Keywords:** Oncogenes, Cancer models

## Abstract

The cell signaling factors EGFR, EphA2, and Ephexin1 are associated with lung and colorectal cancer and play an important role in tumorigenesis. Although the respective functional roles of EGFR and EphA2 are well known, interactions between these proteins and a functional role for the complex is not understood. Here, we showed that Ephexin1, EphA2, and EGFR are each expressed at higher levels in lung and colorectal cancer patient tissues, and binding of EGFR to EphA2 was associated with both increased tumor grade and metastatic cases in both cancer types. Treatment with Epidermal Growth Factor (EGF) induced binding of the RR domain of EGFR to the kinase domain of EphA2, and this binding was promoted by Ephexin1. Additionally, the AKT-mediated phosphorylation of EphA2 (at Ser897) promoted interactions with EGFR, pointing to the importance of this pathway. Two mutations in EGFR, L858R and T790M, that are frequently observed in lung cancer patients, promoted binding to EphA2, and this binding was dependent on Ephexin1. Our results indicate that the formation of a complex between EGFR, EphA2, and Ephexin1 plays an important role in lung and colorectal cancers, and that inhibition of this complex may be an effective target for cancer therapy.

## Introduction

Dysregulation of receptor tyrosine kinases (RTKs) is common in certain cancers. EGFR (Epidermal Growth Factor Receptor) and EphA receptor are the most well-characterized of the RTKs in cancer cells [1–5]. Abnormal and uncontrolled activation of the EGFR pathway correlates with lung and colorectal cancer progression, and EphA receptor overexpression promotes cancer malignancy [3–11]. In addition, EphA2 expression is associated with EGFR inhibitor-resistant lung cancer cells, and combined treatment of EGFR and EphA2 targeted therapy is more effective than monotherapy in colorectal cancer and lung cancer [12, 13]. Therefore, understanding the mechanism by which these two proteins lead to tumorigenesis is an important goal of cancer therapy.

The best characterized EGFR pathway is the Ras/Raf/MEK/ERK signaling cascade. Activation of this pathway regulates the expression of genes involved in tumor progression through the sequential phosphorylation of RAF/MEK/ERK [8–11]. With no bound ligand, EGFR exists as a monomer, but when a ligand such as EGF (epidermal growth factor) binds to the extracellular domain, it forms a dimer and is activated. Activated EGFR may form a homodimer or a heterodimer with another tyrosine kinase receptor [14–18].

EphA2 receptor signaling progresses through one of two pathways, the Ephrin ligand-dependent or independent pathway, depending on the cellular environment. Increased expression of EphA2 is correlated with cancer progression, metastatic spread, and patient survival [1, 2], and overexpression of EphA2 has been shown to specifically increase migration, invasion, metastasis, and angiogenesis [19–24]. Activation of the EGFR/Ras pathway is one of the ways in which EphA2 levels are increased [25–28].

EphA receptors are members of the Rho family of guanosine triphosphatases (GTPase) and interact directly with Ephexin1 (Eph-interacting exchange protein), a member of a subfamily of the Dbl family of guanine nucleotide exchange factors (GEFs) [29, 30]. Ephexin1 also acts as a GEF for RhoA, Rac1, and cdc42 [29], and is highly expressed in the nervous system but rarely expressed in organs other than the liver and kidneys [29, 31]. Ephexin1 has been shown to associate with EphA and is involved in axon outgrowth, synapse remodeling, growth-cone collapse, and motor axon guidance [29, 30, 32–35]. Recently, we found that activating EGFR through EGF treatment or overexpression of mutant K-Ras leads to direct interactions with Ephexin1 [36].

Here we show that depletion of Ephexin1 lowers the tumorigenic effects that are observed when either EGFR or EphA2 is overexpressed. Furthermore, the binding of EGFR and EphA2 to each other correlates to Ephexin1 levels, and interactions between EGFR and EphA2 are associated with an increasingly poor tumor tissue grade.

## Methods

### Cell culture and chemicals

Normal cell lines (IMR90, MRC5, WI38, CCD18co, and CCD841coN) were cultured in MEM medium (Invitrogen, Carlsbad, CA, USA). Lung and colorectal cancer cells (A549, H23, H358, H1299, H1666, HCC-827, H1650, LoVo, HCT15, HCT116) were grown in RPMI-1640 medium (Invitrogen). SK-MES-1, Calu-3, Caco-2, and LS174T cells were cultured in MEM medium. HeLa, HEK293T, SW480, SW620, DLD-1, and HT-29 cells were maintained in Dulbecco’s Modified Eagle Medium (Invitrogen). All cell lines were purchased from the American Type Culture Collection (ATCC, Manassas, VA, USA). All media were supplemented with 10% fetal bovine serum (FBS) and 1% penicillin/streptomycin antibiotic solution. Cells were maintained in 5% CO_2_ in a humidified atmosphere at 37 °C. Plasmids were transiently transfected into mammalian cells using TurboFect (Thermo Scientific, Waltham, MA, USA). When cells were treated with EGF (Sigma-Aldrich, St. Louis, MO, USA), they were first starved for serum overnight (14–16 h) and then EGF (100 ng/ml) was treated for the indicated time.

### Plasmid constructs and cloning

Human EphA1, 2, and EGFR cDNA was amplified from HEK293T cells by RT-PCR using the following primers and cloned into the pCI-neo-Flag or pCI-neo-V5 mammalian expression vectors (Promega, Madison, WI, USA). To prepare serial deletion constructs of EphA2 receptor (∆SAM/PDZ, ∆Kin/SAM/PDZ, and Extra / TM) or EGFR (∆RR and ∆Kin. RR) the PCR products were cloned into the XhoI and NotI sites of pCI-neo-V5 vector. The pCI-neo-Flag-EphA2 mutant (S897D) construct was generated by site-directed mutagenesis (Quikchange II Site-Directed Mutagenesis^®^ kit, Agilent Technologies, Santa Clara, CA, USA). All constructs were verified by DNA sequencing. For cell-free translation, the Flag-EphA2_kinase domain and HA-EGFR_RR domain constructs were cloned into pALiCE01 (ALiCE^®^, Merck). A comprehensive list of all PCR primers used in this study can be found in Supplementary Table S1.

### Immunoblot and immunoprecipitation analysis

Cell extracts were prepared in IP150 lysis buffer (20 mM Tris-HCl pH 7.6, 150 mM NaCl, 0.5% Nonidet P-40, 10% Glycerol) containing protease inhibitors (1 mM Na_2_VO_4_, 10 mM NaF, 2 mM PMSF, 5 μg/ml Leupeptin, 10 μg/ml Aprotinin, 1 μg/ml Pepstatin A) (Roche, Switzerland). Equal amounts of protein were separated by SDS-PAGE and transferred onto PVDF membranes (PALL Life Sciences, USA). Membranes were subsequently incubated with the appropriate primary antibodies overnight at 4 °C, followed by incubation with peroxidase-conjugated secondary antibodies for 1 h at room temperature. Protein bands were visualized using the ECL chemiluminescent detection system (iNtRON Biotechnology, Korea). For immunoprecipitation of protein complexes, cell extracts were precleared with protein G-Sepharose beads (GE Healthcare) and incubated with the appropriate antibodies. Immune complexes were then analyzed by immunoblotting, which was performed using the following antibodies: anti-Ephexin1 and anti-β-actin from Abcam (Cambridge, MA, USA); anti-ERK1/2, antiphospho-ERK1/2 (T202/Y204), anti-Ki67, anti-EGFR, and antiphospho-EphA2 (S897) from Cell Signaling (Danvers, MA, USA); anti-HA, antimyc, anti-V5, and anti-EphA2 from Santa Cruz (Dallas, TX, USA); anti-FLAG (M2) from Sigma-Aldrich; and anti-Ras from BD Biosciences (San Jose, CA, USA).

### Soft agar colony formation assay

Soft agar assays were performed in 6-well plates containing a base layer of 2 ml (at a final concentration of 1X) medium and 0.6% low melting point agarose (Duchefa Biochemie, Netherland). Plates were chilled at 4 °C until the media solidified, and then a growth layer of 2 ml growth agar containing 1 × 10^4^ cells suspended in 1X medium and 0.3% low-melting-point agarose was added. Plates were chilled again at 4 °C until the growth layer congealed. An additional 1 ml of 1X medium without agarose was added on top of the growth layer. Cells were incubated at 37 °C in 5% CO_2_ for approximately 14–21 days, and colonies were stained with 0.005% crystal violet (Sigma-Aldrich) and counted. Images were analyzed using an Olympus microscope (Olympus, Tokyo, Japan) and Image-Pro Plus 4.5 software (Media Cybernetics Inc., Rockville, MD, USA). Assays were performed in triplicate.

### Cell migration assay

In vitro cell migration assays were performed in a 24-well transwell plate with 8 μm polyethylene terephthalate membrane filters (BD Biosciences) separating the lower and upper culture chambers. Cells were grown until they reached sub-confluence (75–80%) and were serum starved for 24 h. After detachment with trypsin, cells were washed with PBS, re-suspended in serum-free medium, and then a cell suspension (2 × 10^4^ cells) was added to the upper chamber. Complete medium was added to the bottom chamber. The cells that had not migrated were removed from the upper surface of the filters using cotton swabs, and the cells that had migrated to the lower surface of the filters were fixed with 4% formaldehyde and stained with 0.2% crystal violet. Images of 3 random 10X magnified fields were captured from each membrane, and the number of migratory cells was counted. The mean of triplicate assays for each experimental condition was used.

### Tumor formation in nude mice

The mice used in this study were 6-week-old male BALB/c nude mice purchased from NARA Biotech (Seoul, Korea). They were housed in our pathogen-free facility and handled in accordance with standard-use protocols and animal welfare regulations. HEK293T cells were harvested and resuspended in PBS. There after 1 × 10^6^ HEK293T cells were injected subcutaneously into the left and right flanks of the mice. Once the tumors became visible, the tumor size was measured every 3 to 4 days using micrometer calipers. Tumor volumes were calculated using the following formula: volume = 0.5 *a* × *b*^2^, where *a* and *b* represent the larger and smaller tumor diameters, respectively. After approximately 3 weeks of injections, mice were humanely sacrificed, and the primary tumors were excised and immediately weighed. All animal studies were reviewed and approved by the Institutional Animal Welfare and Use Committee.

### Immunostaining

Immunohistochemistry was performed on tissue microarrays of lung and colorectal cancer samples. Tissue microarrays from cancer samples of different grades and adjacent normal tissues were purchased from Super Bio Chips (CCA4 and CDA3) (Seoul, South Korea). For immunohistochemistry, heat-induced antigen retrieval was performed using 1X antigen retrieval buffer (pH 9.0) (Abcam) at 95 °C for 15 min. After quenching of endogenous peroxidase and blocking in 3% H_2_O_2_ solution, tissues were incubated with primary anti-Ephexin1 (PA5-52521, Thermo Scientific), anti-EphA2 (sc-924, Santa Cruz) and anti-EGFR (#4267, Cell Signaling) antibodies overnight at 4 °C, followed by incubation with HRP-conjugated secondary antibody for 1 h at room temperature and incubation for 2 min in DAB (3, 3’-Diaminobenzidine). The slides were then counterstained by introducing Harris’s hematoxylin. The intensity of staining was scored from 0 to 4, and extent of staining was scored from 0% to 100%. The final quantitation score for each stain was obtained by multiplying the 2 scores. The slides were analyzed by 2 independent pathologists.

### Proximity ligation assay (PLA)

H1299, HCT116, HEK293T, and HeLa cells were seeded in a 24-well plate and grown for 3 days. The cells were washed with PBS, fixed in 4% paraformaldehyde for 10 min, permeabilized in 0.25% Triton X-100 for 5 min, washed with PBS, and blocked with Duolink™ blocking solution. Tissues were incubated with primary anti-EphA2 [#378-440 (host; mouse), ThermoFisher Scientific] and anti-EGFR [#4267 (host; rabbit), Cell Signaling] antibodies overnight at 4 °C. Slides were incubated in anti-rabbit MINUS and antimouse PLUS PLA probes (Duolink, Sigma-Aldrich) for 1 hr at 37 °C. After a 30 min incubation with ligation buffer and ligase (Duolink™, Sigma-Aldrich) at 37 °C, amplification buffer and polymerase (Duolink™, Sigma-Aldrich) were added, and incubation continued for 120 min at 37 °C. The Proximity Ligation Assay was performed on tissue microarrays of lung cancer of different grades along with adjacent normal tissues that were purchased from Super Bio Chips (CCA4) (Seoul, South Korea). For the assay, heat-induced antigen retrieval was performed using 1X antigen retrieval buffer (pH 9.0) (Abcam) at 95 °C for 15 min and blocked with Duolink™ blocking solution. Tissues were incubated with primary anti-EphA2 and anti-EGFR antibodies overnight at 4 °C. Slides were incubated in anti-rabbit MINUS and anti-mouse PLUS PLA probes (Duolink™, Sigma-Aldrich) for 1 hr at 37 °C. After a 30 min incubation with ligation buffer and ligase (Duolink™, Sigma-Aldrich) at 37 °C, amplification buffer and polymerase (Duolink™, Sigma-Aldrich) were added, and incubation continued for 120 minutes at 37 °C. Stained samples were analyzed with a fluorescence microscope (Nikon, Japan).

### Bioinformatics Analysis using the TCGA and GTEx databases

The Cancer Genome Atlas (TCGA; https://www.cancer.gov/about-nci/organization/ccg/research/structural-genomics/tcga) and Genotype-Tissue Expression program (GTEx; https://commonfund.nih.gov/GTex) were downloaded using the UCSC Xena browser Data Hub (https://xenabrowser.net/hub/). RNA sequencing data measured by Illumina HiSeq (RSEM normalized) was downloaded whenever available. The TCGA mRNA expression of discovery set was transformed into log2 scale and correlation analysis was visualized using the UCSC Xena browser software.

### Statistics

Data were presented as mean ± SEM of three independent experiments and significant differences between groups were assessed by two-tailed paired Student’s *t*-test or Two-way ANOVA using GraphPad Prism (GraphPad Software Inc., CA, USA). Results with a value of *P < 0.05, **P < 0.01, and ***P < 0.001 were considered statistically significant.

## Results

### The overexpression of Ephexin1, EGFR and EphA2 receptors is correlated to lung and colorectal cancers

**Fig. 1:**
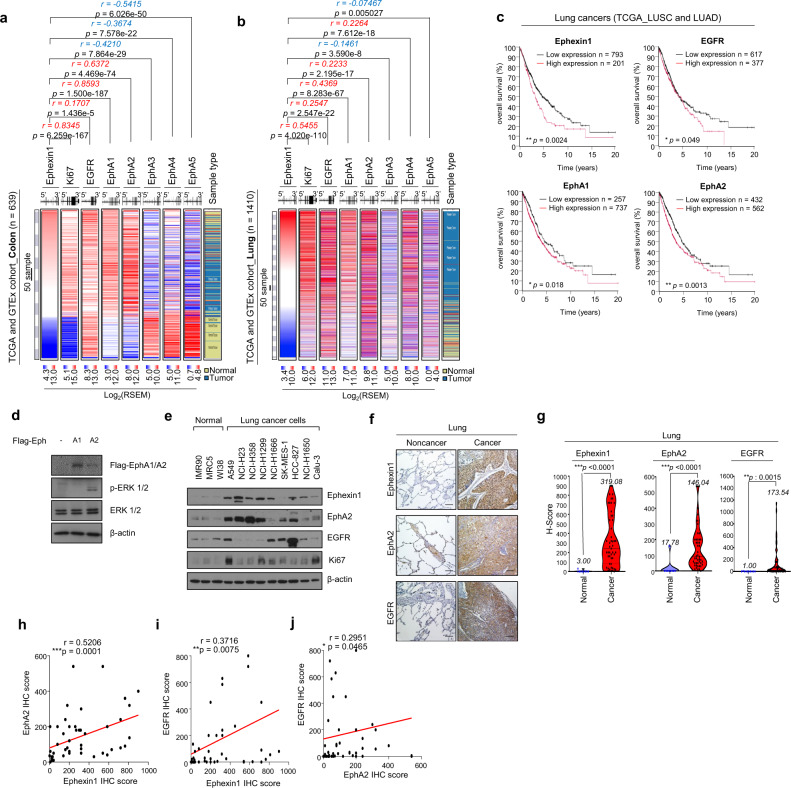
Aberrantly high levels of the cell receptors EGFR, EphA2, and Ephexin1 are associated with lung and colorectal cancers. **a, b** Associations between Ephexin1, Ki67, EGFR, and EphA1-5 levels and tumor tissues were identified by analyzing the TCGA (COAD, COADREAD, LUAD, LUSC) and GTEx datasets. Positive correlations are expressed as mean Spearman r, and p values are for a two-tailed Student’s *t* test. **c** Kaplan-Meier analysis of overall patient survival as related to expression levels of Ephexin1, EGFR, EphA1 and EphA2 in lung cancer patients (TCGA_LUSC and TCGA_LUAD). The *p* values are for a log-rank test. **d** Western blot analysis using the indicated antibodies after transfection of HEK293T cells with either control vector or plasmid expressing Flag-tagged EphA (A1 and A2). **e** Western blot analysis of EGFR, EphA2, Ephexin1 and Ki67 expression from normal and cancer lung cell lines. **f** Immunohistochemical staining for EGFR, EphA2 and Ephexin1 in cancerous (*n* = 40) and corresponding normal tissue (*n* = 10). Scale bar = 100 μm **G** Immunohistochemistry (IHC) score for the data shown in (**f**). Student’s t-test and data are shown as mean ± SEM, **P < 0.01, and ***P < 0.001. **h**–**j** A correlation of IHC levels between Ephexin1 and EphA2 (**h**), Ephexin1 and EGFR (**I**), and EphA2 and EGFR (**j**). Correlated analyses are shown as mean Spearman r, and p values are for a two-tailed Student’s *t* test. *P < 0.05, **P < 0.01, and ***P < 0.001.

Recently, we reported that Ephexin1 is abnormally overexpressed in lung and colorectal cancers and that lack of Ephexin1 effectively suppresses lung and colorectal cancers [36]. Based on previous reports, we hypothesized that there may be an association between EGFR/Ras signaling and EphA/Ephexin1 signaling. To demonstrate this, we analyzed RNA sequence data from The Cancer Genome Atlas (TCGA) and the Genotype-Tissue Expression (GTEx) database and correlated Ephexin1 expression levels with the following proteins: Ki67, EGFR, EphA1, EphA2, EphA3, EphA4, and EphA5. Ki67 is a cancer growth marker and was used as an indicator of accurate classification patterns. When colorectal tissue samples from the databases were analyzed, there was a positive correlation between Ephexin1 and Ki67, EGFR, EphA1, and EphA2, and a negative correlation with EphA3, EphA4, and EphA5 (Fig. 1a). When a lung tissue sample was analyzed, Ephexin1 positively correlated with Ki67, EGFR, EphA1, EphA2, and EphA4, and negatively correlated with EphA3 and EphA5 (Fig. 1b). Since EGFR, EphA1, and EphA2 positively correlated with Ephexin1 in both colon and lung tissues, we focused on these three proteins and analyzed the survival rates of lung cancer and colorectal cancer patients relative to the expression levels of all four proteins. In an analysis of survival rates among lung cancer patients, the higher the expression of Ephexin1, EGFR, EphA1, and EphA2, the lower the rate (Fig. 1c). In contrast, the lower the expression of EphA3, EphA4, and EphA5, the worse the survival rate (Supplementary Fig. S1a). Similarly, for colorectal cancer patients, higher expression of Ephexin1 and EphA2 correlated to worse survival rates, and lower expression of EphA3 correlated to worse survival rates (Supplementary Fig. S1b, c). Therefore, we predicted that EphA1 or EphA2 were the most likely of the EphA receptor family members to be relevant to Ephexin1 and EGFR signaling.

To confirm this, we tested whether the overexpression of EphA1 and EphA2 led to predictable changes in the EGFR/Ras pathway. When Flag-tagged versions of each were individually overexpressed in HEK293T cells, ERK phosphorylation was higher in the presence of Flag-tagged EphA2 but not in the presence of Flag-tagged EphA1 (Fig. 1d). Consistent with these results, the expression of EphA2, as well as Ephexin1 and EGFR, was aberrantly upregulated in lung cancer cell lines compared to normal human primary lung fibroblasts (Fig. 1e) and was also much higher in colorectal cancer cell lines than normal human colon cells (Supplementary Fig. S1d). Next, we analyzed changes in protein expression in normal and cancer tissues using IHC. Again, Ephexin1, EGFR, and EphA2 were at significantly higher levels in lung and colon cancer tissues than in normal lung and colon tissues (Fig. 1f-g and Supplementary S1e-f). Using IHC data in lung cancer patient tissues, we looked for correlations between protein levels and observed similar results to the RNA sequencing data analysis. There was a statistically significant positive correlation between Ephexin1-EphA2 (*r* = 0.5206, *p* = 0.0001), Ephexin1-EGFR (*r* = 0.3716, *p* = 0.0075) and EphA2-EGFR (*r* = 0.2951, *p* = 0.0465) (Fig. 1h–j). Together, these results suggest a link between lung and colorectal cancers and the expression of Ephexin1, EphA2 and EGFR, and the possibility that these proteins interact with each other, either directly or indirectly.

### The kinase domain of EphA2 and the RR domain of EGFR are important for protein interactions

**Fig. 2:**
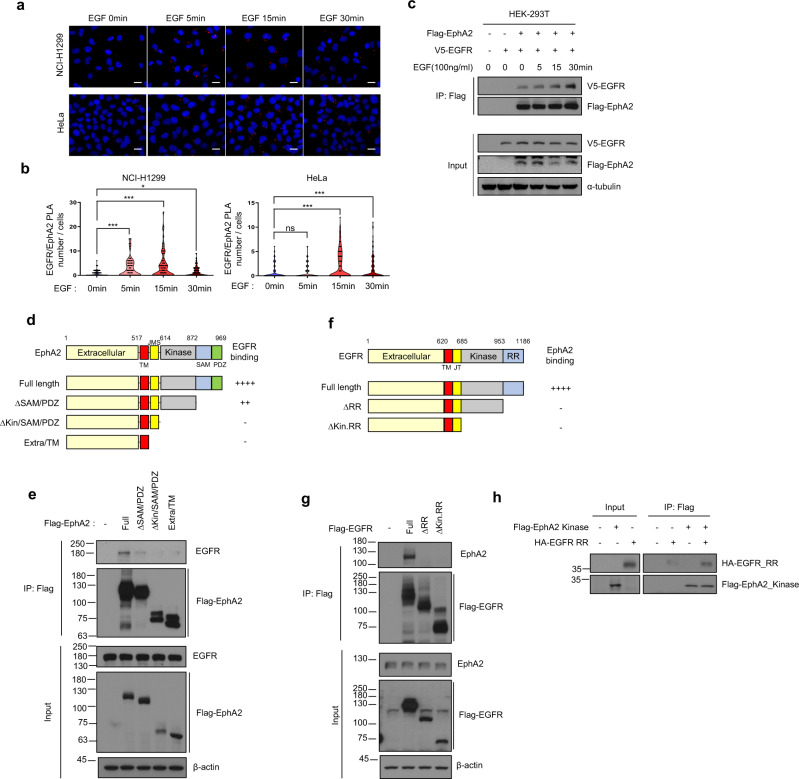
Ephexin1 mediates interactions between the kinase domain of EphA2 and the RR domain of EGFR. **a** Proximity ligation assay (PLA) was used to detect a complex between EphA2 and EGFR in H1299 and HeLa cells that were starved for serum for 16 h and then treated with EGF (100 ng/ml). Localization of EphA2 and EGFR together is shown in red. The cells were counterstained with DAPI (blue) to visualize the nuclei. Scale bar = 20 μm **b** Quantification of the PLA data shown in (**a**). **c** Immunoprecipitation (IP) with an anti-Flag antibody of protein extracts from HEK-293T cells co-transfected with Flag-tagged EphA2 and V5-tagged EGFR, that were treated with EGF (100 ng/ml) after a 16 h serum starvation. Western blot analysis was performed with indicated antibodies. **d** Schematic representation of wild type EphA2 and a series of deletion mutants. A summary of the degree to which each interacts with EGFR is shown to the right. **e** Lysates from HEK293T cells transfected with Flag-tagged wild type EphA2 or deletion mutants were immunoprecipitated with anti-Flag antibody and subjected to western blot analysis with the indicated antibodies. **f** Schematic representation of wild type EGFR and a series of deletion mutants. A summary of the degree to which each interacts with EphA2 is shown to the right. **g** Lysates from HEK293T cells transfected with Flag-tagged wild type EGFR or deletion mutants was immunoprecipitated with anti-Flag antibody and subjected to western blot analysis with indicated antibodies. **h** Pulldown assay to measure the binding of Flag-tagged EphA2_kinase domain with HA-tagged EGFR_RR domain. Immunoprecipitation was carried out using the anti-Flag antibody.

It has been reported that EphA2 binds to EGFR in human cancer cell lines [25]. However, a role for direct interactions between these two receptors was not well understood. We looked for conditions in which the interaction between EGFR and EphA2 was increased in cancer cells and analyzed the binding site between the two receptors as a way to identify a functional role. First, we treated cells with growth factor, because growth factors are known to lead to re-localization and conformational changes of several receptors in the cell membrane [37, 38]. When H1299 and HeLa cells were treated with EGF, the number of foci, as analyzed through a PLA, indicating interactions between EGFR and EphA2, was increased (Fig. 2a, b and Supplementary Fig. S2a, b). Similarly, when HEK293T cells overexpressing Flag-EphA2 and V5-EGFR were treated with EGF and then immunoprecipitated using an anti-Flag antibody, interactions between EGFR and EphA2 were measurably increased (Fig. 2c).

Identifying the specific regions within the two proteins that interact is very helpful in predicting their role in the cell [39, 40]. Therefore, we analyzed the potential interacting regions in EphA2 and EGFR using Flag-tagged constructs of four different mutants of each. Full-length and deletion mutants of Flag-EphA2 were transfected into HEK293T cells and then immunoprecipitated from cell lysates using an anti-Flag antibody. When the SAM/PDZ domain was deleted, the binding of EphA2 to EGFR was significantly reduced compared to the full length, and when the kinase domain of EphA2 was deleted, EGFR did not bind (Fig. 2d, e). Likewise, when three different deletion mutations of Flag-tagged EGFR were transfected into HEK293T cells and immunoprecipitated from cell lysates with anti-Flag antibody, EphA2 interactions were abolished when the RR domain of EGFR was deleted (Fig. 2f, g). To demonstrate direct binding of the RR domain of EGFR to the EphA2 kinase domain, we synthesized a Flag-EphA2 kinase domain and a RR domain of HA-EGFR using a cell-free expression system. After combining the two, immunoprecipitation was performed using an anti-Flag antibody, and interactions between these domains was confirmed (Fig. 2h).

### Low levels of Ephexin1 prevents interactions between EphA2 and EGFR

**Fig. 3:**
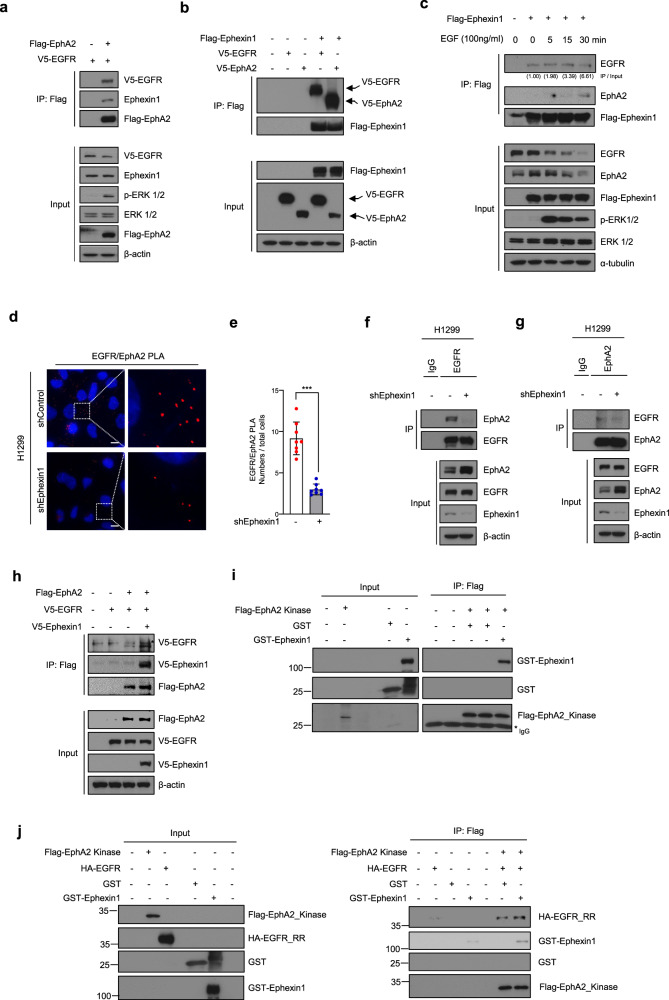
Ephexin1 is required for the binding between EGFR and EphA2. **a** Immunoprecipitation with anti-Flag antibody of protein extracts from HEK293T cells co-transfected with Flag-tagged EphA2 and V5-tagged EGFR. Western blot analysis was carried out with the indicated antibodies. **b** Immunoprecipitation with anti-Flag antibody of extracts from HEK293T cells co-transfected with Flag-tagged Ephexin1, V5-tagged EGFR, and/or V5-tagged EphA2 plasmids. Western blot analysis was carried out with the indicated antibodies. **c** Immunoprecipitation with anti-Flag antibody of extracts from HEK293T cells transfected with Flag-tagged Ephexin1 and treated with EGF (100 ng/ml) after 16 h of serum starvation. Western blot analysis was carried out with the indicated antibodies. **d** Proximity ligation assay (PLA) of EphA2 and EGFR in either shControl or shEphexin1-H1299 cells showing the location of both proteins (red). The cells were counterstained with DAPI (blue) to visualize the nuclei. Scale bar = 10 um. **e** Quantification of the Proximity ligation assay (PLA) shown in (**d**). Data are shown as mean ± SD. ***P < 0.001 as determined through a two-tailed Student’s *t* test. **f, g** Lysates from shControl or shEphexin1 H1299 cells were immunoprecipitated with anti-EGFR antibody or anti-EphA2 antibody. western blot analysis with the indicated antibodies. **H** Immunoprecipitation analysis with anti-Flag antibody and analysis with the indicated antibodies of HEK293T cells co-transfected with Flag-tagged EphA2, V5-tagged EGFR, and/or V5-tagged Ephexin1 plasmids. **I** Pulldown assays using an anti-Flag antibody of the Flag-tagged EphA2_Kinase domain with GST or GST-Ephexin1. **j** Pulldown assays using an anti-Flag antibody of the Flag-tagged-EphA2_kinase domain, HA-tagged-EGFR_RR domain, and GST-Ephexin1 as indicated.

Ephexin1 has been shown to associate with the kinase domain of EphA4 [29]. Since the kinase domain of EphA2 is very similar to that of EphA4 (76.5% identity and 95.0% positive by FASTA: https://www.ebi.ac.uk/Tools/sss/), we predicted that Ephexin1 would also bind to EphA2 and influence associations with EGFR. To test this hypothesis, we transfected HEK293T cells with Flag-EphA2 and V5-EGFR, and immunoprecipitated with anti-Flag antibodies. Flag-EphA2 simultaneously bound to both EGFR and Ephexin1 (Fig. 3a). Correspondingly, EGFR, EphA2 and Ephexin1 bound to each other at endogenous levels in H1299 and HCT116 cells (Supplementary Fig. S3a, b). Immunofluorescence of H1299 and HCT116 cells confirmed that Ephexin1, EphA2, and EGFR localize to the same site in the cell (Supplementary Fig. S3c). These results demonstrated interactions between EphA2 and EGFR and between EphA2 and Ephexin1. We next investigated whether Ephexin1 affects the binding of EphA2 to EGFR. To explore this, HEK293T cells were transiently transfected with V5-tagged EGFR and V5-tagged EphA2 along with or without Flag-tagged Ephexin1, and then Ephexin1 was immunoprecipitated using an anti-Flag antibody. Indeed, we observed that Ephexin1 increased the binding of EphA2 to EGFR (Fig. 3b). Correspondingly, treatment with EGF led to an increase in the interactions between EGFR and EphA2, as measured by immunoprecipitation with Flag-Ephexin1 (Fig. 3c). These results suggest that Ephexin1, EphA2, and EGFR form a single complex.

Based on these results, we tested the effect of Ephexin1 deficiency on the interaction of EGFR with EphA2. In a previous report, we performed target off effect verification by testing two Ephexin1 shRNA and sgRNA sequences in various cells [36]. The change of EGFR and EphA2 interaction according to Ephexin1 deficiency was tested in H1299 cells using PLA assay. Ephexin1 deficiency in H1299 cells decreased the number of PLA-foci compared to control cells (Fig. 3d, e). Similarly, in HEK293T and HCT116 cells, Ephexin1 deficiency significantly reduced the number of PLA-foci of EGFR-EphA2 (Supplementary Fig. S3d, e). Correspondingly, when EGFR or EphA2 was immunoprecipitated using an anti-EGFR and anti-EphA2 antibodies in Ephexin1-deficient H1299 and HCT116 cells, the interaction between EphA2 and EGFR was significantly reduced compared to the control group (Fig. 3f-g and Supplementary Fig. S3f). Conversely, overexpression of V5-Ephexin1 increased the interaction between EphA2 and EGFR (Fig. 3h). The pull-down analysis showed that the Flag-EphA2 kinase domain and GST-Ephexin1 bind to each other (Fig. 3i). The binding of the Flag-EphA2 kinase domain with the HA-tagged RR domain of EGFR was stronger when added together with GST-Ephexin1 (Fig. 3j). Taken together, these results suggest that Ephexin1 may play an important role in the interaction between EphA2 and EGFR.

### AKT promotes interactions between EphA2 and both EGFR and Ephexin1

**Fig. 4:**
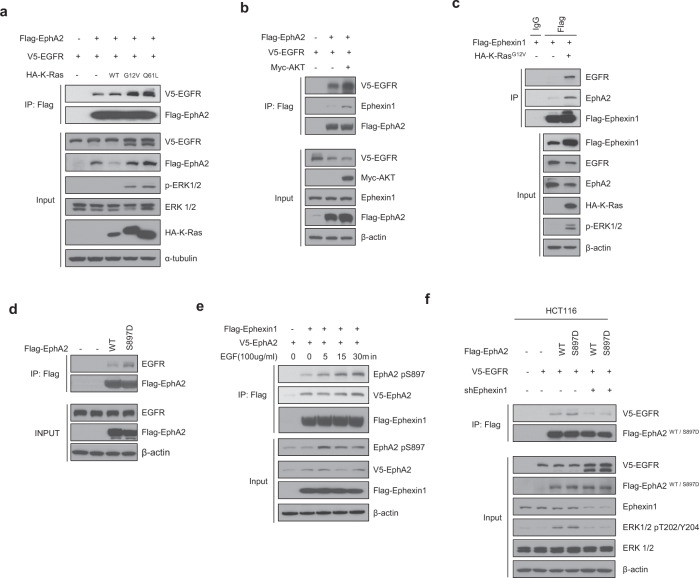
AKT mediates phosphorylation of EphA2 at Ser897 which then promotes binding to EGFR in the presence of Ephexin1. For each experiment, immunoprecipitation was carried out with protein extracts from the indicated HEK293T cell lines using anti-Flag antibody and then the precipitate was analyzed by western blot analysis using antibodies indicated in the panels. **a** Extracts from cells co-transfected with Flag-EphA2, V5-EGFR and HA-K-Ras (WT, G12V, and Q61L) plasmids. **b** Extracts from cells transfected with Flag-tagged EphA2, V5-tagged EGFR along with or without Myc-tagged myr-AKT (constitutively active). **c** Extracts from cells co-transfected with Flag-tagged Ephexin1 with or without HA-tagged K-Ras^G12V^ plasmids. **d** Extracts from cells transfected with Flag-tagged EphA2 WT or S897D plasmids. **e** Extracts from cells co-transfected with Flag-tagged Ephexin1 and V5-tagged EphA2 that were treated with EGF (100 ng/ml) after 16 h serum starvation. Western blot analysis was carried out using anti-EphA2 pSer897 antibody. **f** Extracts from cells co-transfected with Flag-tagged EphA2 WT or S897D, V5-tagged EGFR with or without shEphexin1 plasmids.

AKT is a representative downstream effector of the EGFR/Ras signaling pathway and is known to increase the oncogenic function of EphA2 [41]. Therefore, we predicted that the binding of EGFR to EphA2 would be regulated by RAS/AKT signaling. To test this, we transfected Flag-EphA2, V5-EGFR and HA-K-Ras (WT, G12V, or Q61L) into HEK293T cells, and then immunoprecipitated with an anti-Flag antibody. Binding between EphA2 and EGFR was increased when either of the K-Ras mutants was overexpressed (Fig. 4a). Likewise, when Myc-AKT myr (constitutively active) was overexpressed, binding between EphA2 and EGFR was increased (Fig. 4b). The next question was whether activated Ras signaling also influenced the binding of these proteins. Indeed, when Flag-Ephexin1 and HA-KRas^G12V^ (constitutively active Ras) were expressed in HEK293T cells and immunoprecipitated with an anti-Flag antibody, increased binding of Ephexin1, EphA2 and EGFR was observed, confirming a role for Ras (Fig. 4c).

### AKT-induced phosphorylation of the Ser897 site of EphA2 promotes interactions between EGFR and Ephexin1

Activated AKT mediates phosphorylation at the Ser897 site of EphA2, which has been shown to then be associated with cancer progression [41]. Since deletion of the SAM/PDZ domain of EphA2 reduced binding to EGFR, and the SAM domain of EphA2 has Ser897 (Fig. 2d, e), we predicted that this phosphorylation event would promote binding of EGFR. To establish this, we transfected HEK293T cells with either WT EphA2 (Flag-EphA2 WT) or a phosphomimetic mutant (Flag-EphA2 S897D), and immunoprecipitated with an anti-Flag antibody. Indeed, more significant binding to EGFR was observed for the phosphomimetic mutant (S897D) than for WT (Fig. 4d). To determine whether binding of Ephexin1 to EphA2 followed the same pattern, we transfected HEK293T cells with Flag-Ephexin1 and V5-EphA2, treated with EGF, and immunoprecipitated with an anti-Flag antibody. EGF treatment led to an increase in phosphorylation of the Ser897 site of EphA2 and also an increase in the binding of Flag-Ephexin1 to EphA2, particularly to phosphorylated EphA2 (Ser897) (Fig. 4e). To further explore the role of Ephexin1 in the AKT-mediated binding of EphA2 to EGFR we overexpressing Flag-EphA2 (WT or S897D mutant) and V5-EGFR in Ephexin1-deficient HCT116 cells and control cells and immunoprecipitated with an anti-Flag antibody. There was no increase in binding between the EphA2 S897D mutant and EGFR in the absence of Ephexin1 (Fig. 4f). Taken together, these results indicate that activation of AKT by EGFR/Ras induces the phosphorylation of EphA2 at Ser897 and promotes interactions between EGFR and EphA2. The increased binding of EGFR to EphA2 in the presence of Ephexin1 suggests that Ephexin1 acts as an intermediate mediator of this interaction.

### Ephexin1 deficiency reduces tumor growth and migration ability induced by EphA2 and EGFR

**Fig. 5:**
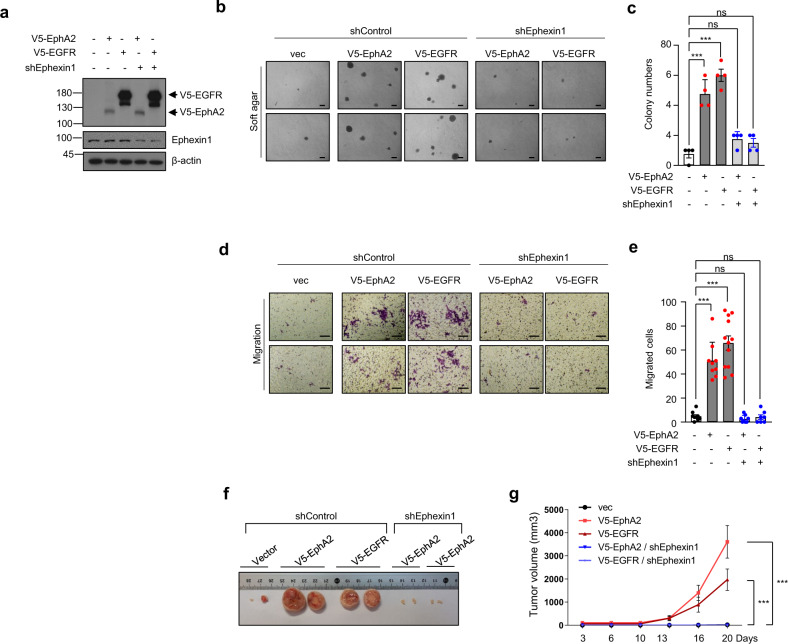
In the absence of Ephexin1, the tumorigenesis normally induced when EphA2 or EGFR is overexpressed is decreased. **a** Western blot analysis using the indicated antibodies of extracts from HEK293T cells stably transfected with V5-tagged EphA2 or V5-tagged EGFR, with or without the shEphexin1 plasmid. **b** Anchorage-independent growth assay of cells with V5-tagged EphA2 or V5-tagged EGFR, with or without the shEphexin1 plasmid. Representative images are shown. Scale bar = 100 μm. **c** Quantification of the anchorage-independent growth assay shown in (**b**). *p* values are for a two-tailed Student’s *t* test, ***P < 0.001. ns, not significant. **d** Migration assay of cells with V5-tagged EphA2 or V5-tagged EGFR, with or without the shEphexin1 plasmid. Representative images are shown. Scale bar = 100 μm. **e** Quantification of the migration assay shown in (**d**). *p* values are for a two-tailed Student’s *t* test, ***P < 0.001. ns, not significant. **f** Tumor growth of xenografts in nude mice (*n* = 4) derived from empty-vector, V5-tagged EphA2 or V5-tagged EGFR along with or without shEphexin1 plasmids in HEK293T cells. Representative images of the tumors at the endpoint of the experiment are shown. **g** Growth curves of mammary tumors after implantation. Data are shown as mean ± SEM. p values are for a two-way ANOVA, ***P < 0.001.

Overexpression of EphA2 and EGFR is known to increase tumorigenesis [26, 42–51]. In this study, we show that Ephexin1 may play an important role in the binding of EphA2 to EGFR. (Figs. 3, 4) Therefore, we predicted that a deficiency in Ephexin1 would also decrease the oncogenic effects of EphA2 and EGFR. Because HEK293T cells are frequently used in tumorigenesis and xenograft models of multiple gene transfection [52–56], we tested anchorage-independent growth and migration of cells overexpressing V5-EphA2 or V5-EGFR in either Ephexin1-deficient HEK293T cells or control cells. In control cells, anchorage-independent growth and migration were significantly increased when either V5-EphA2 or V5-EGFR were overexpressed. However, in Ephexin1-deficient cells, there was no change in either cell behavior. (Fig. 5a-e) To confirm that the tumor suppression observed in vitro could be recapitulated in vivo, equal numbers of cells were injected subcutaneously into the right and left flanks of BALB/c nude mice and tumor volumes were measured every 3-4 days. When V5-EphA2 and V5-EGFR were overexpressed, tumors collected at 20 days post-injection were significantly larger than tumors that developed with control cells. However, in an Ephexin1-deficient cell background, overexpression of the two proteins did not lead to an increase in tumor size (Fig. 5f, g). These results suggest that the levels of Ephexin1 regulate the oncogenic functions of EphA2 and EGFR.

### In lung and colorectal cancer, a complex of EphA2 and EGFR is clinically relevant

**Fig. 6:**
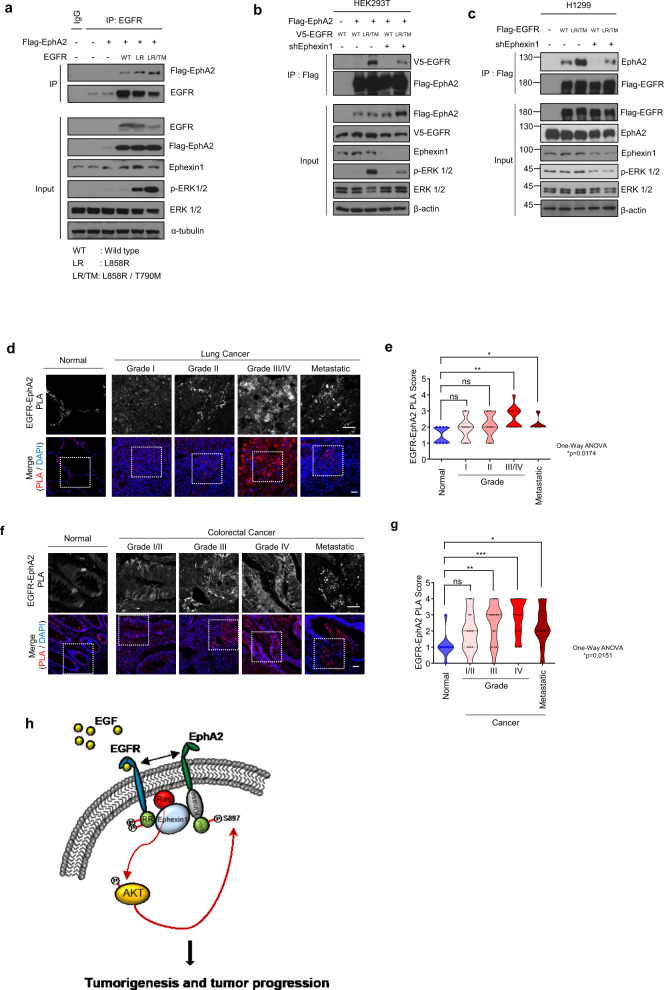
Interactions between EGFR and EphA2 are associated with poor prognosis in lung cancers. **a** Immunoprecipitation with anti-EGFR antibody and western blot analysis with the indicated antibodies of HEK293T cells co-transfected with Flag-tagged EphA2 and EGFR (WT, L858R, or L858R / T1790M mutant) plasmids. **b** Immunoprecipitation with anti-Flag antibody and western blot analysis with the indicated antibodies of extracts from HEK293T cells co-transfected with Flag-tagged EphA2 and V5-tagged EGFR (WT or L858R / T790M), with or without the shEphexin1 plasmid. **c** Immunoprecipitation with anti-Flag antibody and western blot analysis with the indicated antibodies of extracts from H1299 cells transfected with Flag-tagged EGFR (WT or L858R / T790M), with or without the shEphexin1 plasmid. **d** Proximity ligation assay (PLA) was performed to identify interactions between EGFR and EphA2 (red) in grade I (*n* = 13), grade II (*n* = 14), and grade III/V (*n* = 13), and metastatic (*n* = 10) lung cancer tissues and their corresponding normal tissues (*n* = 9). DAPI (blue) was used as a counterstain to visualize the nuclei. Scale bar = 50 μm. **e** Quantification of the PLA data shown in (**d**)**. f** Proximity ligation assay (PLA) was performed to identify interactions between EGFR and EphA2 (red) in grade I/II (*n* = 13), grade III (*n* = 15), grade IV (*n* = 12), and metastatic (*n* = 10) colorectal cancer tissues and their corresponding normal tissues (*n* = 9). DAPI (blue) was used as a counterstain to visualize the nuclei. Scale bar = 50 μm. **G** Quantification of the PLA data shown in (**f**)**. h** A model to describe the major mechanisms behind the tumorigenic effects of the EGFR-Ephexin-EphA2 complex.

To investigate the importance of Ephexin1, EGFR and EphA2 in lung and colorectal cancer patient tissues, we analyzed the TMA database for lung and colorectal tissues composed of carcinomas and metastatic tumors of different grades to determine the expression levels of each of the three proteins. EGFR and EphA2 were expressed at significantly higher levels in cancer tissues than in normal tissues, and levels increased to significant degrees with advancing tumor cell grade (Supplementary Fig. S4a-d). Point mutations in EGFR are frequently found in patients with lung cancer of this type, and those known to activate EGFR include L858R and T790M [57]. To confirm that EphA2 binds to mutated forms of EGFR and is relevant in these lung cancers, HEK293T cells transfected with Flag-EphA2 and EGFR (WT, L858R, or L858R/T790M) were immunoprecipitated with anti-EGFR antibody. The results showed that, not only did EphA2 bind to the EGFR mutants (L858R and L858R/T790M), but it also bound more tightly than to wild-type EGFR (Fig. 6a). Consistent with our previous results, when binding was assessed in an Ephexin1-deficient background by transfecting Ephexin1-deficient HEK293T cells and control cells with Flag-EphA2 and V5-EGFR (WT or L858R/T790M) and immunoprecipitating with anti-Flag antibody, no increase in binding was observed. (Fig. 6b) Likewise, in H1299 lung cancer cells, there was no increase in binding of EphA2 to the EGFR mutant when Ephexin1 was absent (Fig. 6c). We then used PLA analysis to look for in vivo interactions between EGFR and EphA2 in both normal and cancerous cells using lung and colorectal cancer patient tissues. There was a significantly higher number of foci representing the protein complex in cancer tissues than in normal tissues, and the number of foci increased significantly with worsening tumor cell grade and metastatic tumors status (Fig. 6d-g). These results show that an interaction between EGFR and EphA2 is clinically relevant in human lung and colorectal cancers, and provide the basis for the development of targeted anticancer drugs to inhibit this interaction.

## Discussion

In the present study, we found that the expression of EGFR, EphA2, and Ephexin1 was upregulated and correlated to lung and colorectal cancers. Interestingly, anchorage-independent growth, migration, and xenograft tumor growth were increased when either EGFR or EphA2 was overexpressed, but these effects were dependent on the presence of Ephexin1. These results point to an important role for Ephexin1 in regulating the tumorigenicity of EGFR and EphA2 expression.

We show that EGFR, EphA2, and Ephexin1 bind directly with each other to form a single complex. Overexpressed Ephexin1 increased the binding of EphA2 to EGFR, whereas a lack of Ephexin1 had the opposite effect. The kinase function of the EphA receptor plays a role in the functional regulation of downstream effectors [58], but EphA has other distinct functions as well [59]. The kinase domain of EphA4, a receptor that is structurally very similar to EphA2, was not required for binding to Ephexin1 [29, 33]. However, our results showed that the kinase domain of EphA2 is required for interactions with the RR domain of EGFR in an Ephexin1-dependent manner. The activation of EGFR by growth factor signaling is known to lead to autophosphorylation of the RR domain, which then triggers downstream signal transduction events [60–63].

Activation of K-Ras activates AKT, which then phosphorylates Ser897 of EphA2 and results in increased cancer proliferation [41, 64, 65]. The SAM domain of EphA2, which includes Ser897, is not important for the kinase activity but does influence protein-protein interactions [59, 66]. We show that overexpression of either Ephexin1 or mutant K-Ras increased interactions between EphA2 and EGFR. In addition, we found that an EphA2 phosphomimetic mutant (pSer897) increased this interaction, suggesting that phosphorylation is relevant. However, again, no increase in interactions between EGFR and EphA2 S897D mutant was observed in Ephexin1-deficient cells.

Among non-small-cell lung cancer (NSCLC) patients that are non-smokers, 15-50% have EGFR mutations and 15-25% have K-Ras mutations, both of which play an important role in tumor progression and metastasis [67, 68]. Therefore, many researchers are developing cancer treatments targeting EGFR, K-Ras, Ras-downstream effectors, and EphA2. For example, Gefitinib and Erlotinib, EGFR-tyrosine kinase inhibitors (EGFR-TKI), prolonged progression-free survival compared to conventional platinum-based chemotherapy in a randomized controlled phase 3 clinical trial [69, 70]. Osimertinib, a third-generation EGFR-TKI, is currently undergoing phase 3 clinical trials (Phase3, NCT02296125). BT5528 is a bicyclic peptide that binds to EphA2, and is currently undergoing phase 1 clinical trials (Phase 1, NCT04180371), and EphA2 monoclonal antibody DS-8895a has completed phase 1 clinical trials (Phase 1, NCT02252211). However, despite these excellent therapeutic results, current cancer cell-targeted therapies have increased the survival rate of patients, but long-term administration often leads to recurrence and resistance [71–74]. For example, long-term administration of an inhibitor that specifically blocks the K-Ras mutant protein has resulted in resistance and the observable recovery of phospho-ERK and phospho-EGFR levels [75–77]. In addition, re-activation of Ras signaling is induced when there is an increase in the expression of RTK [13, 78–80]. Our results suggest that when resistance to cancer treatments targeting Ras signaling arises, the observed re-activation of Ras may have been due to an increase in the levels of the EphA2 / EGFR / Ephexin1 complex.

Since the EGFR/RAF/MEK/ERK pathway is essential for normal cellular processes, indiscriminately inhibiting this pathway would target both normal cells and cancer cells. A more effective treatment would target proteins that are differentially expressed in cancer cells. Ephexin1 is rarely expressed in normal cells besides neurons [29, 31]. Therefore, a combination treatment that includes an Ephexin1-specific inhibitor and targeted inhibitors of EGFR, EphA2, or K-Ras is predicted to improve the effectiveness of cancer treatment.

In conclusion, Ephexin1 is proposed as an effective target protein for the therapy of tumors in lung and colorectal cancers since it plays an important role in regulating interactions between EGFR and EphA2.

## Supplementary information

Supplementary Figures and Table

Original Data File

Checklist

## Data Availability

All data generated or analyzed in this study are included in this paper and can be obtained from the corresponding author according to formal requirement.

## References

[1] Wykosky J, Gibo DM, Stanton C, Debinski W (2005). EphA2 as a novel molecular marker and target in glioblastoma multiforme. Mol Cancer Res.

[2] Macrae M, Neve RM, Rodriguez-Viciana P, Haqq C, Yeh J, Chen C (2005). A conditional feedback loop regulates Ras activity through EphA2. Cancer Cell.

[3] Ullrich A, Coussens L, Hayflick JS, Dull TJ, Gray A, Tam AW (1984). Human epidermal growth factor receptor cDNA sequence and aberrant expression of the amplified gene in A431 epidermoid carcinoma cells. Nature.

[4] Di Fiore PP, Pierce JH, Fleming TP, Hazan R, Ullrich A, King CR (1987). Overexpression of the human EGF receptor confers an EGF-dependent transformed phenotype to NIH 3T3 cells. Cell.

[5] Moscatello DK, Montgomery RB, Sundareshan P, McDanel H, Wong MY, Wong AJ (1996). Transformational and altered signal transduction by a naturally occurring mutant EGF receptor. Oncogene.

[6] Pedersen MW, Tkach V, Pedersen N, Berezin V, Poulsen HS (2004). Expression of a naturally occurring constitutively active variant of the epidermal growth factor receptor in mouse fibroblasts increases motility. Int J Cancer.

[7] Chaffanet M, Chauvin C, Laine M, Berger F, Chedin M, Rost N (1992). EGF receptor amplification and expression in human brain tumours. Eur J Cancer.

[8] Buday L, Downward J (1993). Epidermal growth factor regulates p21ras through the formation of a complex of receptor, Grb2 adapter protein, and Sos nucleotide exchange factor. Cell.

[9] Downward J (2003). Targeting RAS signalling pathways in cancer therapy. Nat Rev Cancer.

[10] Marais R, Light Y, Paterson HF, Marshall CJ (1995). Ras recruits Raf-1 to the plasma membrane for activation by tyrosine phosphorylation. EMBO J.

[11] Howe LR, Leevers SJ, Gomez N, Nakielny S, Cohen P, Marshall CJ (1992). Activation of the MAP kinase pathway by the protein kinase raf. Cell.

[12] Martini G, Cardone C, Vitiello PP, Belli V, Napolitano S, Troiani T (2019). EPHA2 Is a Predictive Biomarker of Resistance and a Potential Therapeutic Target for Improving Antiepidermal Growth Factor Receptor Therapy in Colorectal Cancer. Mol Cancer Ther.

[13] Amato KR, Wang S, Tan L, Hastings AK, Song W, Lovly CM (2016). EPHA2 Blockade Overcomes Acquired Resistance to EGFR Kinase Inhibitors in Lung Cancer. Cancer Res.

[14] Lemmon MA, Bu Z, Ladbury JE, Zhou M, Pinchasi D, Lax I (1997). Two EGF molecules contribute additively to stabilization of the EGFR dimer. EMBO J.

[15] Holbro T, Civenni G, Hynes NE (2003). The ErbB receptors and their role in cancer progression. Exp Cell Res.

[16] Gilbertson RJ, Perry RH, Kelly PJ, Pearson AD, Lunec J (1997). Prognostic significance of HER2 and HER4 coexpression in childhood medulloblastoma. Cancer Res.

[17] Osaki A, Toi M, Yamada H, Kawami H, Kuroi K, Toge T (1992). Prognostic significance of co-expression of c-erbB-2 oncoprotein and epidermal growth factor receptor in breast cancer patients. Am J Surg.

[18] Xia W, Lau YK, Zhang HZ, Xiao FY, Johnston DA, Liu AR (1999). Combination of EGFR, HER-2/neu, and HER-3 is a stronger predictor for the outcome of oral squamous cell carcinoma than any individual family members. Clin Cancer Res.

[19] Mudali SV, Fu B, Lakkur SS, Luo M, Embuscado EE, Iacobuzio-Donahue CA (2006). Patterns of EphA2 protein expression in primary and metastatic pancreatic carcinoma and correlation with genetic status. Clin Exp Metastasis.

[20] Kinch MS, Moore MB, Harpole DH (2003). Predictive value of the EphA2 receptor tyrosine kinase in lung cancer recurrence and survival. Clin Cancer Res.

[21] Herrem CJ, Tatsumi T, Olson KS, Shirai K, Finke JH, Bukowski RM (2005). Expression of EphA2 is prognostic of disease-free interval and overall survival in surgically treated patients with renal cell carcinoma. Clin Cancer Res.

[22] Ireton RC, Chen J (2005). EphA2 receptor tyrosine kinase as a promising target for cancer therapeutics. Curr Cancer Drug Targets.

[23] Wykosky J, Debinski W (2008). The EphA2 receptor and ephrinA1 ligand in solid tumors: function and therapeutic targeting. Mol Cancer Res.

[24] Zhuang G, Brantley-Sieders DM, Vaught D, Yu J, Xie L, Wells S (2010). Elevation of receptor tyrosine kinase EphA2 mediates resistance to trastuzumab therapy. Cancer Res.

[25] Larsen AB, Pedersen MW, Stockhausen MT, Grandal MV, van Deurs B, Poulsen HS (2007). Activation of the EGFR gene target EphA2 inhibits epidermal growth factor-induced cancer cell motility. Mol Cancer Res.

[26] Zelinski DP, Zantek ND, Stewart JC, Irizarry AR, Kinch MS (2001). EphA2 overexpression causes tumorigenesis of mammary epithelial cells. Cancer Res.

[27] Zantek ND, Walker-Daniels J, Stewart J, Hansen RK, Robinson D, Miao H (2001). MCF-10A-NeoST: a new cell system for studying cell-ECM and cell-cell interactions in breast cancer. Clin Cancer Res.

[28] Andres AC, Zuercher G, Djonov V, Flueck M, Ziemiecki A (1995). Protein tyrosine kinase expression during the estrous cycle and carcinogenesis of the mammary gland. Int J Cancer.

[29] Shamah SM, Lin MZ, Goldberg JL, Estrach S, Sahin M, Hu L (2001). EphA receptors regulate growth cone dynamics through the novel guanine nucleotide exchange factor ephexin. Cell.

[30] Sahin M, Greer PL, Lin MZ, Poucher H, Eberhart J, Schmidt S (2005). Eph-dependent tyrosine phosphorylation of ephexin1 modulates growth cone collapse. Neuron.

[31] Rodrigues NR, Theodosiou AM, Nesbit MA, Campbell L, Tandle AT, Saranath D (2000). Characterization of Ngef, a novel member of the Dbl family of genes expressed predominantly in the caudate nucleus. Genomics.

[32] Fu WY, Chen Y, Sahin M, Zhao XS, Shi L, Bikoff JB (2007). Cdk5 regulates EphA4-mediated dendritic spine retraction through an ephexin1-dependent mechanism. Nat Neurosci.

[33] Frank CA, Pielage J, Davis GW (2009). A presynaptic homeostatic signaling system composed of the Eph receptor, ephexin, Cdc42, and CaV2.1 calcium channels. Neuron.

[34] Shi L, Butt B, Ip FC, Dai Y, Jiang L, Yung WH (2010). Ephexin1 is required for structural maturation and neurotransmission at the neuromuscular junction. Neuron.

[35] Chang CJ, Chang MY, Chou SY, Huang CC, Chuang JY, Hsu TI (2018). Ephexin1 Is Required for Eph-Mediated Limb Trajectory of Spinal Motor Axons. J Neurosci.

[36] Kim J, Jeon YJ, Lim SC, Ryu J, Lee JH, Chang IY (2021). Akt-mediated Ephexin1-Ras interaction promotes oncogenic Ras signaling and colorectal and lung cancer cell proliferation. Cell Death Dis.

[37] Roepstorff K, Grandal MV, Henriksen L, Knudsen SL, Lerdrup M, Grovdal L (2009). Differential effects of EGFR ligands on endocytic sorting of the receptor. Traffic.

[38] Schlessinger J (2000). Cell signaling by receptor tyrosine kinases. Cell.

[39] Rao VS, Srinivas K, Sujini GN, Kumar GN (2014). Protein-protein interaction detection: methods and analysis. Int J Proteom.

[40] Schreiber G. Protein–Protein Interaction Interfaces and their Functional Implications. 2020;1–24.

[41] Miao H, Li DQ, Mukherjee A, Guo H, Petty A, Cutter J (2009). EphA2 mediates ligand-dependent inhibition and ligand-independent promotion of cell migration and invasion via a reciprocal regulatory loop with Akt. Cancer Cell.

[42] Kinch MS, Carles-Kinch K (2003). Overexpression and functional alterations of the EphA2 tyrosine kinase in cancer. Clin Exp Metastasis.

[43] Fang WB, Ireton RC, Zhuang G, Takahashi T, Reynolds A, Chen J (2008). Overexpression of EPHA2 receptor destabilizes adherens junctions via a RhoA-dependent mechanism. J Cell Sci.

[44] Brantley-Sieders DM, Zhuang G, Hicks D, Fang WB, Hwang Y, Cates JM (2008). The receptor tyrosine kinase EphA2 promotes mammary adenocarcinoma tumorigenesis and metastatic progression in mice by amplifying ErbB2 signaling. J Clin Invest.

[45] Zhang H, Shi JH, Jiang H, Wang K, Lu JY, Jiang X (2018). ZBTB20 regulates EGFR expression and hepatocyte proliferation in mouse liver regeneration. Cell Death Dis.

[46] Destro A, Ceresoli GL, Falleni M, Zucali PA, Morenghi E, Bianchi P (2006). EGFR overexpression in malignant pleural mesothelioma. An immunohistochemical and molecular study with clinico-pathological correlations. Lung Cancer.

[47] Ning T, Peng Z, Li S, Qu Y, Zhang H, Duan J (2017). miR-455 inhibits cell proliferation and migration via negative regulation of EGFR in human gastric cancer. Oncol Rep..

[48] Song W, Hwang Y, Youngblood VM, Cook RS, Balko JM, Chen J (2017). Targeting EphA2 impairs cell cycle progression and growth of basal-like/triple-negative breast cancers. Oncogene.

[49] Syed N, Barbhuiya MA, Pinto SM, Nirujogi RS, Renuse S, Datta KK (2015). Phosphotyrosine profiling identifies ephrin receptor A2 as a potential therapeutic target in esophageal squamous-cell carcinoma. Proteomics.

[50] Huang J, Xiao D, Li G, Ma J, Chen P, Yuan W (2014). EphA2 promotes epithelial-mesenchymal transition through the Wnt/beta-catenin pathway in gastric cancer cells. Oncogene.

[51] Huang C, Yuan W, Lai C, Zhong S, Yang C, Wang R (2020). EphA2-to-YAP pathway drives gastric cancer growth and therapy resistance. Int J Cancer.

[52] Long L, He JZ, Chen Y, Xu XE, Liao LD, Xie YM (2018). Riboflavin Depletion Promotes Tumorigenesis in HEK293T and NIH3T3 Cells by Sustaining Cell Proliferation and Regulating Cell Cycle-Related Gene Transcription. J Nutr.

[53] Lee HL, Chen CC, Baasov T, Ron Y, Dougherty JP (2011). Post-transcriptionally regulated expression system in human xenogeneic transplantation models. Mol Ther.

[54] Hamid T, Malik MT, Kakar SS (2005). Ectopic expression of PTTG1/securin promotes tumorigenesis in human embryonic kidney cells. Mol Cancer.

[55] Debeb BG, Zhang X, Krishnamurthy S, Gao H, Cohen E, Li L (2010). Characterizing cancer cells with cancer stem cell-like features in 293T human embryonic kidney cells. Mol Cancer.

[56] Voce P, D’Agostino M, Moretti S, Sponziello M, Rhoden K, Calcinaro F (2011). Sunitinib inhibits tumor vascularity and growth but does not affect Akt and ERK phosphorylation in xenograft tumors. Oncol Rep.

[57] Wu SG, Yu CJ, Tsai MF, Liao WY, Yang CH, Jan IS (2013). Survival of lung adenocarcinoma patients with malignant pleural effusion. Eur Respir J.

[58] Egea J, Nissen UV, Dufour A, Sahin M, Greer P, Kullander K (2005). Regulation of EphA 4 kinase activity is required for a subset of axon guidance decisions suggesting a key role for receptor clustering in Eph function. Neuron.

[59] Kullander K, Mather NK, Diella F, Dottori M, Boyd AW, Klein R (2001). Kinase-dependent and kinase-independent functions of EphA4 receptors in major axon tract formation in vivo. Neuron.

[60] Downward J, Waterfield MD, Parker PJ (1985). Autophosphorylation and protein kinase C phosphorylation of the epidermal growth factor receptor. Effect on tyrosine kinase activity and ligand binding affinity. J Biol Chem.

[61] Margolis BL, Lax I, Kris R, Dombalagian M, Honegger AM, Howk R (1989). All autophosphorylation sites of epidermal growth factor (EGF) receptor and HER2/neu are located in their carboxyl-terminal tails. Identification of a novel site in EGF receptor. J Biol Chem.

[62] Okabayashi Y, Kido Y, Okutani T, Sugimoto Y, Sakaguchi K, Kasuga M (1994). Tyrosines 1148 and 1173 of activated human epidermal growth factor receptors are binding sites of Shc in intact cells. J Biol Chem.

[63] Sorkin A, Mazzotti M, Sorkina T, Scotto L, Beguinot L (1996). Epidermal growth factor receptor interaction with clathrin adaptors is mediated by the Tyr974-containing internalization motif. J Biol Chem.

[64] Paraiso KH, Das Thakur M, Fang B, Koomen JM, Fedorenko IV, John JK (2015). Ligand-independent EPHA2 signaling drives the adoption of a targeted therapy-mediated metastatic melanoma phenotype. Cancer Disco.

[65] Miao H, Gale NW, Guo H, Qian J, Petty A, Kaspar J (2015). EphA2 promotes infiltrative invasion of glioma stem cells in vivo through cross-talk with Akt and regulates stem cell properties. Oncogene.

[66] Kullander K, Klein R (2002). Mechanisms and functions of Eph and ephrin signalling. Nat Rev Mol Cell Biol.

[67] Capella G, Cronauer-Mitra S, Pienado MA, Perucho M (1991). Frequency and spectrum of mutations at codons 12 and 13 of the c-K-ras gene in human tumors. Environ Health Perspect.

[68] Jorge SE, Kobayashi SS, Costa DB (2014). Epidermal growth factor receptor (EGFR) mutations in lung cancer: preclinical and clinical data. Braz J Med Biol Res.

[69] Mitsudomi T, Morita S, Yatabe Y, Negoro S, Okamoto I, Tsurutani J (2010). Gefitinib versus cisplatin plus docetaxel in patients with non-small-cell lung cancer harbouring mutations of the epidermal growth factor receptor (WJTOG3405): an open label, randomised phase 3 trial. Lancet Oncol.

[70] Rosell R, Carcereny E, Gervais R, Vergnenegre A, Massuti B, Felip E (2012). Erlotinib versus standard chemotherapy as first-line treatment for European patients with advanced EGFR mutation-positive non-small-cell lung cancer (EURTAC): a multicentre, open-label, randomised phase 3 trial. Lancet Oncol.

[71] Holohan C, Van Schaeybroeck S, Longley DB, Johnston PG (2013). Cancer drug resistance: an evolving paradigm. Nat Rev Cancer.

[72] Garraway LA, Janne PA (2012). Circumventing cancer drug resistance in the era of personalized medicine. Cancer Disco.

[73] Brown R, Curry E, Magnani L, Wilhelm-Benartzi CS, Borley J (2014). Poised epigenetic states and acquired drug resistance in cancer. Nat Rev Cancer.

[74] Gottesman MM (2002). Mechanisms of cancer drug resistance. Annu Rev Med.

[75] Misale S, Fatherree JP, Cortez E, Li C, Bilton S, Timonina D (2019). KRAS G12C NSCLC Models Are Sensitive to Direct Targeting of KRAS in Combination with PI3K Inhibition. Clin Cancer Res.

[76] Molina-Arcas M, Moore C, Rana S, van Maldegem F, Mugarza E, Romero-Clavijo P, et al. Development of combination therapies to maximize the impact of KRAS-G12C inhibitors in lung cancer. Sci Transl Med. 2019;11:eaaw7999. 10.1126/scitranslmed.aaw7999 PMC6764843 31534020

[77] Xue JY, Zhao Y, Aronowitz J, Mai TT, Vides A, Qeriqi B (2020). Rapid non-uniform adaptation to conformation-specific KRAS(G12C) inhibition. Nature.

[78] Sun C, Bernards R (2014). Feedback and redundancy in receptor tyrosine kinase signaling: relevance to cancer therapies. Trends Biochem Sci.

[79] Duncan JS, Whittle MC, Nakamura K, Abell AN, Midland AA, Zawistowski JS (2012). Dynamic reprogramming of the kinome in response to targeted MEK inhibition in triple-negative breast cancer. Cell.

[80] Koch H, Busto ME, Kramer K, Medard G, Kuster B (2015). Chemical Proteomics Uncovers EPHA2 as a Mechanism of Acquired Resistance to Small Molecule EGFR Kinase Inhibition. J Proteome Res.

