# Supplementary material for: Interactions between EGFR and EphA2 promote tumorigenesis through the action of Ephexin1
Source: Cell Death Dis. 2022 Jun 6;13(6):528. doi: 10.1038/s41419-022-04984-6 (PMC9170705; doi:10.1038/s41419-022-04984-6)
Supplement: Supplementary file 1 — Supplementary Figures and Table [file 41419_2022_4984_MOESM1_ESM.pdf]

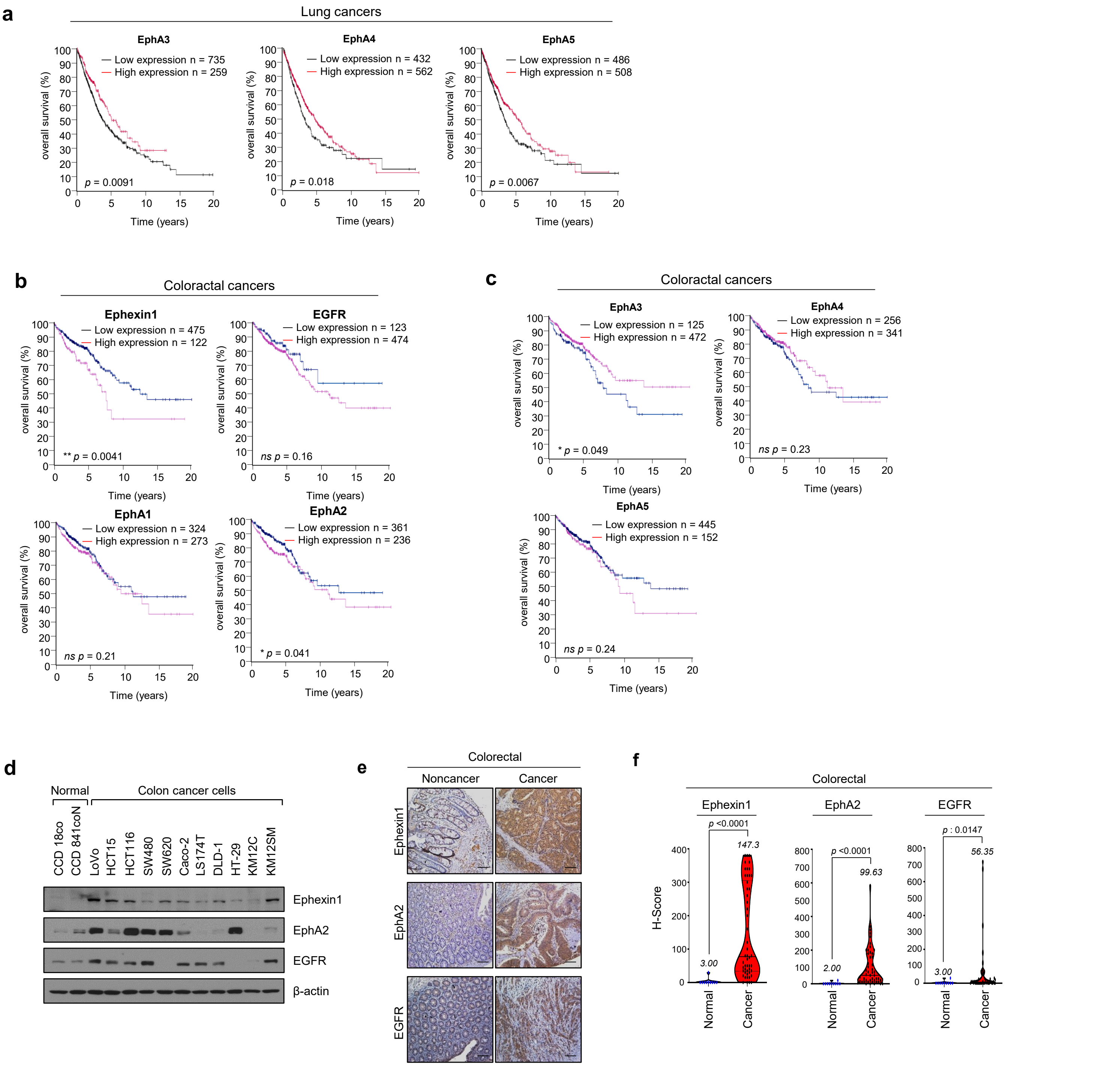

Supplementary Fig. S1 Expression levels of Ephexin1, EphA2, and EGFR correlate to patient survival rates in lung and colorectal cancer. A Kaplan-Meier analysis of overall patient survival rates according to EphA3, EphA4 and EphA5 expression levels in lung cancer patients (TCGA_LUAD and TCGA_LUSC). p values are for a log-rank test. **B, C** Kaplan-Meier analysis of overall survival according to Ephexin1, EGFR, EphA1-A5 expression levels in colorectal cancer patients (TCGA_COAD and TCGA_COADREAD). p values are for a log-rank test. **D** Western blot analysis of EGFR, EphA2 and Ephexin1 expression from colorectal normal and cancer cell lines using the indicated antibodies. **E** Immunohistochemical staining for EGFR, EphA2 and Ephexin1 in cancerous (n = 40) and corresponding normal tissues (n = 10). Scale bar = 100 μm **F** Immunohistochemistry (IHC) scores for the data shown in (**E**). Student's t-test and data are shown as mean ± SEM, $**P < 0.01$, and $***P < 0.001$.

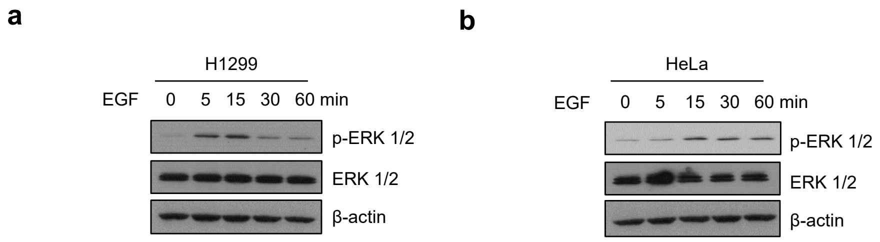

Supplementary Fig. S2 Analysis of ERK phosphorylation by EGF treatment. A, B H1299 (**A**) and HeLa (**B**) cells were treated with EGF (100 ng/ml) for the indicated amounts of time after 16 h of serum starvation. Cell lysates were western blot with indicated antibodies.

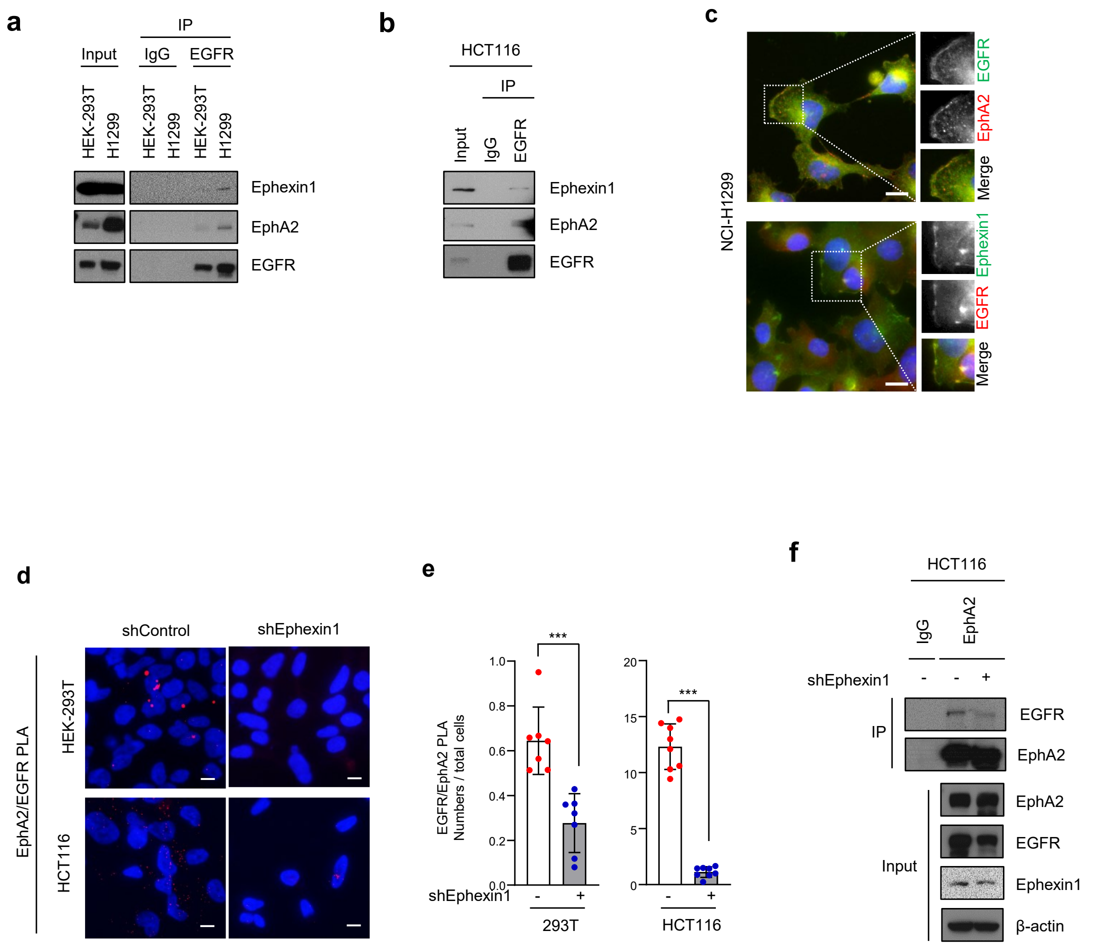

Supplementary Fig. S3 A deficiency in Ephexin1 inhibits the binding of EphA2 to EGFR. A Lysates from HEK293T and H1299 cells were immunoprecipitated with anti-EGFR antibody and subjected to western blot analysis with the indicated antibodies. **B** Immunoprecipitation with anti-EGFR antibody of extracts from HCT116 cells. Western blot analysis was carried out with the indicated antibodies. **C** Immunofluorescent staining with EphA2 (Green) and EGFR (Red) of fixed H1299 and HCT116 cells. Nuclei were stained with Hoechst 33258 (blue). Scale bars = 10 μm. **D** Proximity ligation assay (PLA) of EphA2 and EGFR (interacting proteins shown in red) in shControl or shEphexin1 cells. The cells were counterstained with DAPI (blue) to visualize the nuclei. Scale bar = 10um. **E** Quantification of the data shown in (D) Data are shown as mean ± SEM. ***$P <$ 0.001. **F** Lysates from shControl or shEphexin1 HCT116 cells were immunoprecipitated with anti-EphA2 antibody and subjected to western blot analysis with the indicated antibodies.

Sup. Fig. 4

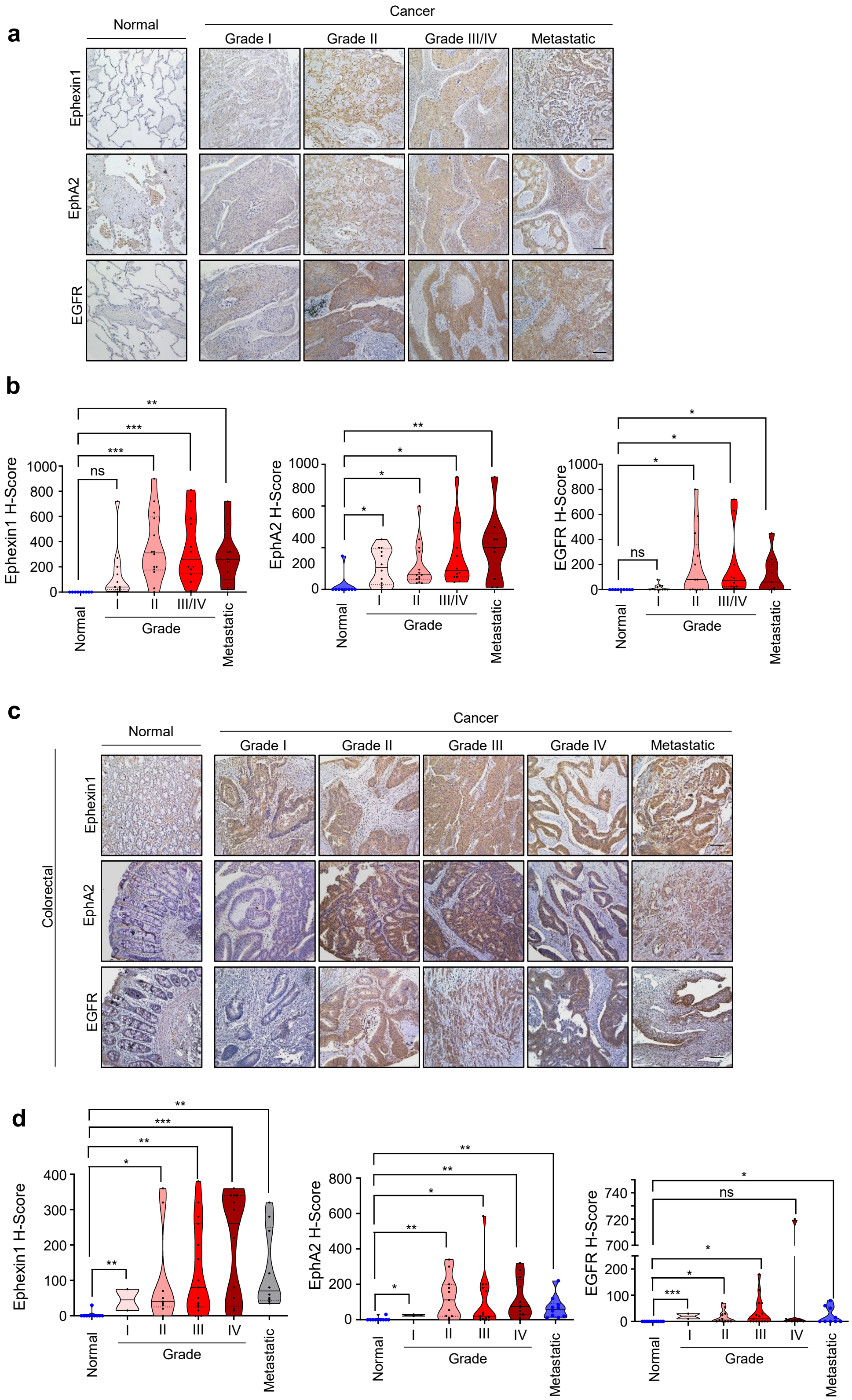
a
Normal
Cancer
Grade I
Grade II
Grade III/IV
Metastatic
Ephexin1
EphA2
EGFR
b
Ephexin1 H-Score
EphA2 H-Score
EGFR H-Score
ns
Normal
Grade
I
II
III/IV
Metastatic
c
Normal
Cancer
Grade I
Grade II
Grade III
Grade IV
Metastatic
Colorectal
Ephexin1
EphA2
EGFR
d
Ephexin1 H-Score
EphA2 H-Score
EGFR H-Score
ns
Normal
Grade
I
II
III
IV
Metastatic

Supplementary Figure S4 Increased expression of Ephexin1, EGFR, and EphA2 is associated with poor prognosis in lung and colorectal cancer. A Immunohistochemistry staining to evaluate Ephexin1, EphA2, and EGFR expression in grade I (n = 11), grade II (n = 14), grade III/V (n = 12), and metastatic (n = 9) lung cancer tissues and their corresponding normal tissues (n = 10). Hematoxylin was used as the counterstain. Scale bar = 100 μm.

B Quantification of the expression scores of Ephexin1, EphA2 and EGFR in lung and colorectal tissues from the data shown in (A). Student's t-test and data are shown as mean ± SEM, ns, not significant, * $P < 0.05$, **$P < 0.01$, and ***$P < 0.001$. **C** Immunohistochemistry staining to evaluate Ephexin1, EphA2, and EGFR expression in grade I (n = 3), grade II (n = 9), grade III (n = 15), grade V (n = 12), and metastatic (n = 10) lung and colorectal cancer tissues and their corresponding normal tissues (n = 10). Hematoxylin was used as the counterstain. Scale bar = 100 μm. **D** Quantification of the expression scores of Ephexin1, EphA2 and EGFR in lung and colorectal tissues from the data shown in (A). Student's t-test and data are shown as mean ± SEM, ns, not significant, * $P < 0.05$, **$P < 0.01$, and ***$P < 0.001$.

Supplementary Table S1. List of primer sequences for cloning.

Genes	Forward primer sequence	Reverse primer sequence
Flag or V5-EphA1 Full length	5'-AAAAGAATTCAATGGAGCGGCGCTGGC-3'	5'-AAAAGCGGCCGCTCAGTCCTTGAATCCCTGAATACTG-3'
Flag or V5-EphA2 Full length	5'-AAAACTCGAGATGGAGCTCCAGGCAGCC-3'	5'-AAAACCCGGGTCAGATGGGGATCCCCACA-3'
Flag or V5-EphA2 ΔSAM	5'-AAAACTCGAGATGGAGCTCCAGGCAGCC-3'	5'-AAAACCCGGGTCAGTCCAGGATGCTGACGATGTCA-3'
Flag or V5-EphA2 ΔKin, SAM	5'-AAAACTCGAGATGGAGCTCCAGGCAGCC-3'	5'-AAAACCCGGGTCAGACACAGGATGGATGGATCTCG-3'
Flag or V5-EphA2 Extra / TM	5'-AAAACTCGAGATGGAGCTCCAGGCAGCC-3'	5'-AAAACCCGGGTCAGATAAAGAAGCCAACTCCTGCCA-3'
Flag or V5-EGFR F ull length	5'-AAAACTCGAGATGCGACCCTCCGGGAC-3'	5'-AAAAGCGGCCGCTCATGCTCCAATAAATTCACTGC-3'
Flag or V5-EGFR ΔRR	5'-AAAACTCGAGATGCGACCCTCCGGGAC-3'	5'-AAAAGCGGCCGCTTATATCATCCAGCACTTGACCATGA-3'
Flag or V5-EGFR ΔKin, RR	5'-AAAACTCGAGATGCGACCCTCCGGGAC-3'	5'-AAAAGCGGCCGCTTACCTCTCCTGCAGCAGCCTC-3'
Flag-EphA2 kinase	5'-AAAAACATGTCTGACTACAAAGACGATGACGAC AAGAAGTTCACTACCGAGATCCATCCAT-3'	5'-AAAAGGTACCTCAAAAGTCAGCCAGGGTCTTGAGG-3'
HA-EGFR_RR	5'-AAAACCATGGCTTACCCATACGATGTTCCA GATTACGCTCTCCCTCAGCCACCCATATGTA-3'	5'-AAAAGGTACCTCATGCTCCAATAAATTCACTGCT-3'
GST-Ephexin1	5'-AAAACTCGAGATGGAGACCAGGGAATCTGAAG-3'	5'-AAAAGCGGCCGCTCATTGCCGATTCCGGC-3'
Flag or V5-EGFR L858R	5'-CCCAGCAGTTTGGCCCGCCCAAAATCTGTGA-3'	5'-TCACAGATTTTGGGCGGGCCAAACTGCTGGG-3'
Flag or V5-EGFR T790M	5'-GCATGAGCTGCATGATGAGCTGCACGGTGG-3'	5'-CCACCGTGCAGCTCATCATGCAGCTCATGC-3'
EphA2 S897D	5'-CGAGCCGCTCGTGTCGGGGAGCCGGATA-3'	5'-TATCCGGCTCCCCGACACGAGCGGCTCG-3'
