# Supplementary material for: Interactions between EGFR and EphA2 promote tumorigenesis through the action of Ephexin1
Source: Cell Death Dis. 2022 Jun 6;13(6):528. doi: 10.1038/s41419-022-04984-6 (PMC9170705; doi:10.1038/s41419-022-04984-6)

Figure 1d

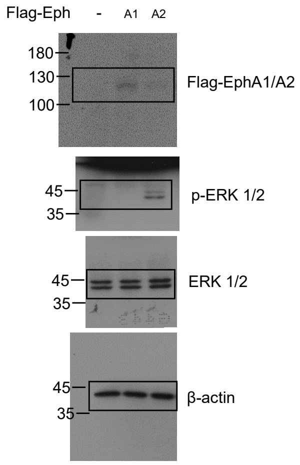

Figure 1e

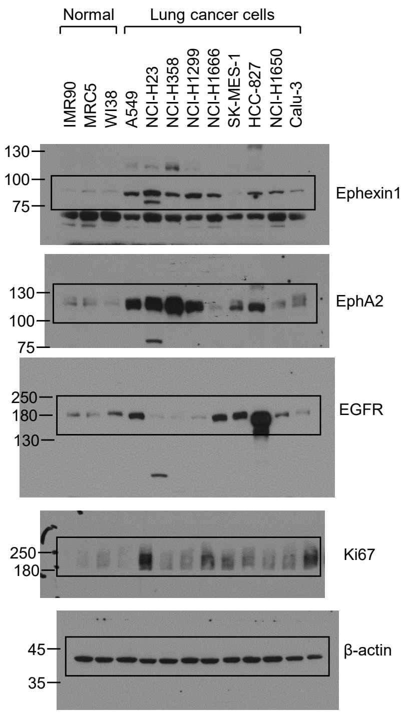

Figure 2e

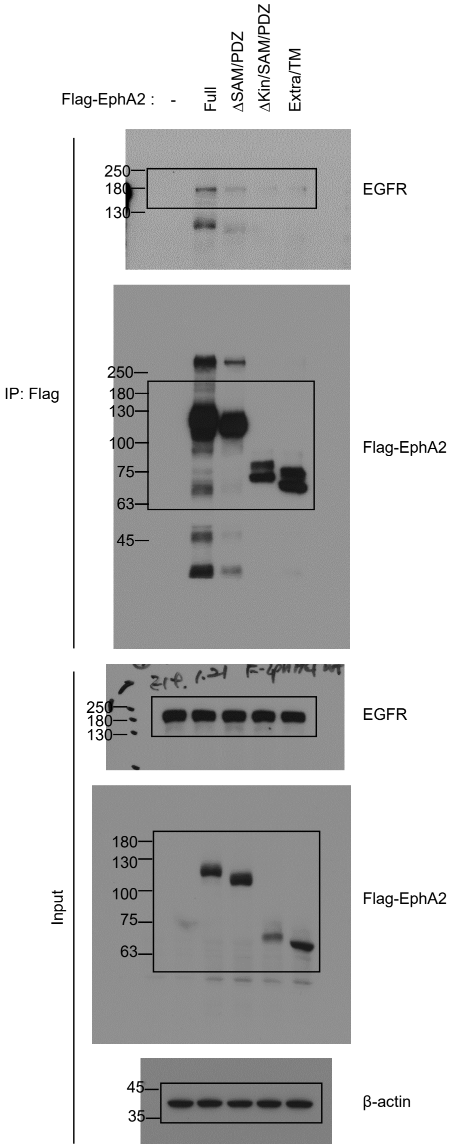

Figure 2g

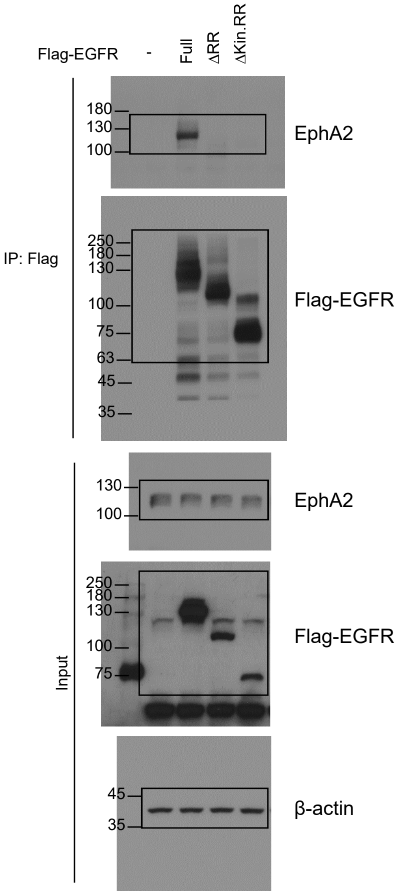

Figure 2h

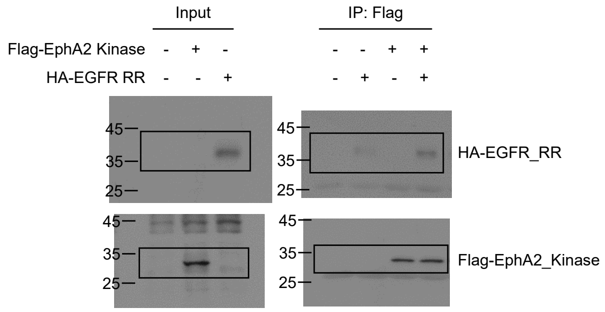

Figure 3a

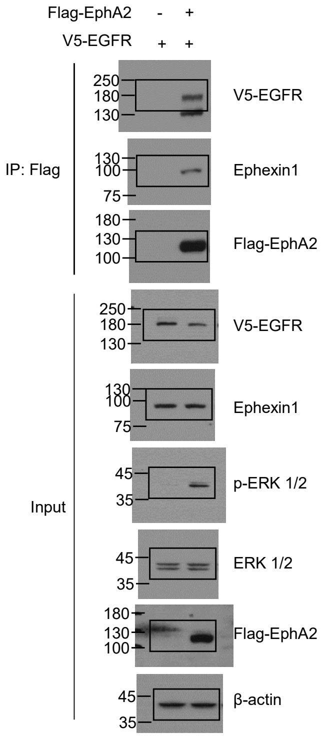

Figure 3b

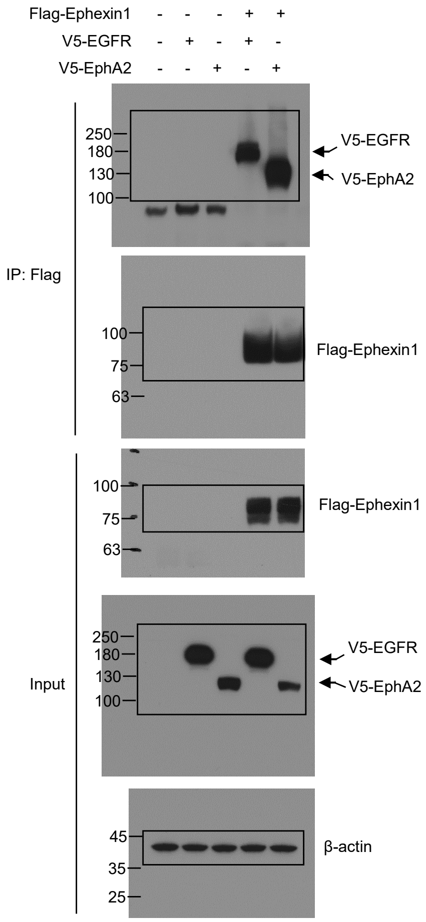

Figure 3c

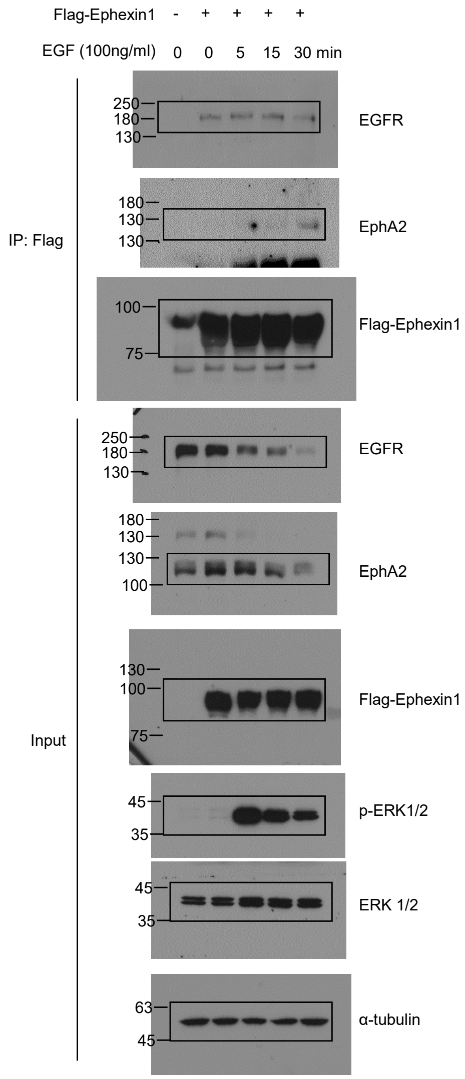

Figure 3i

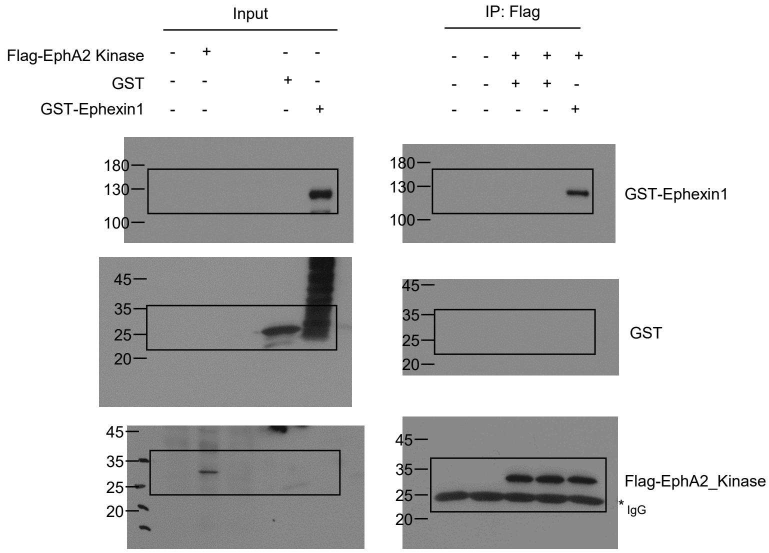

Figure 3f

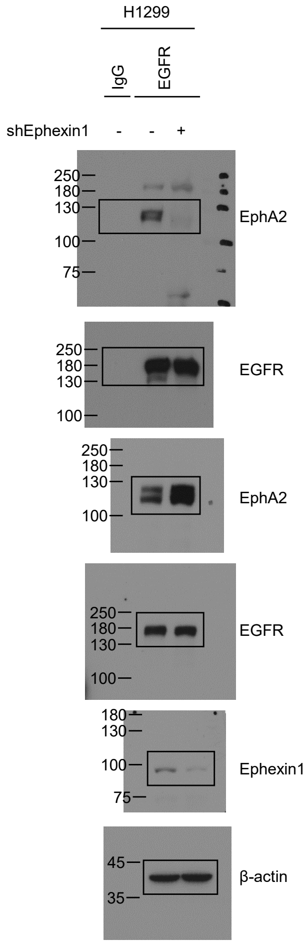

Figure 3g

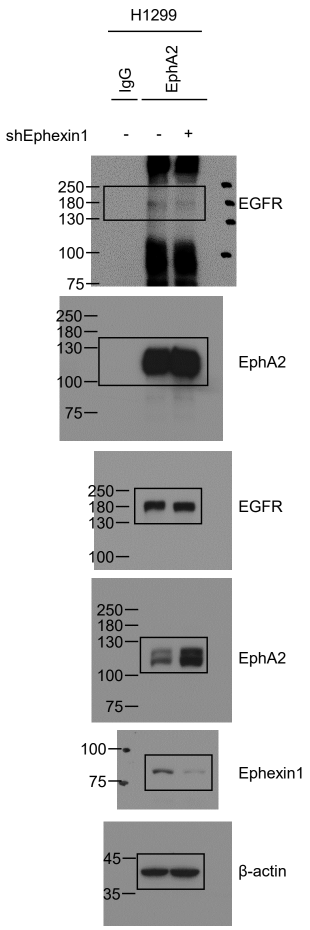

Figure 3h

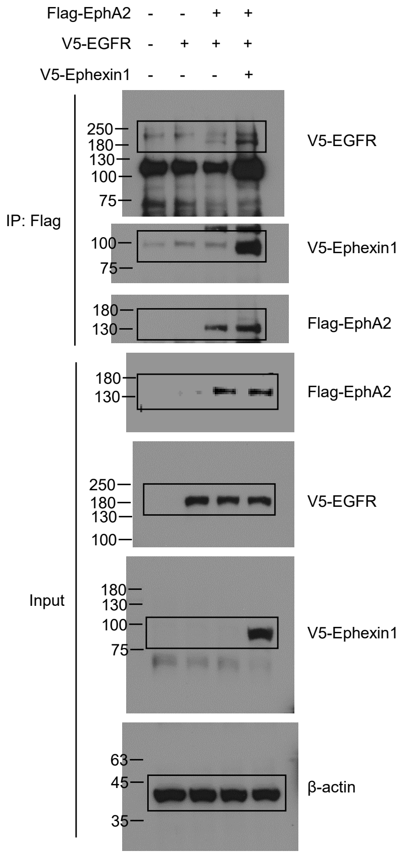

Figure 3j

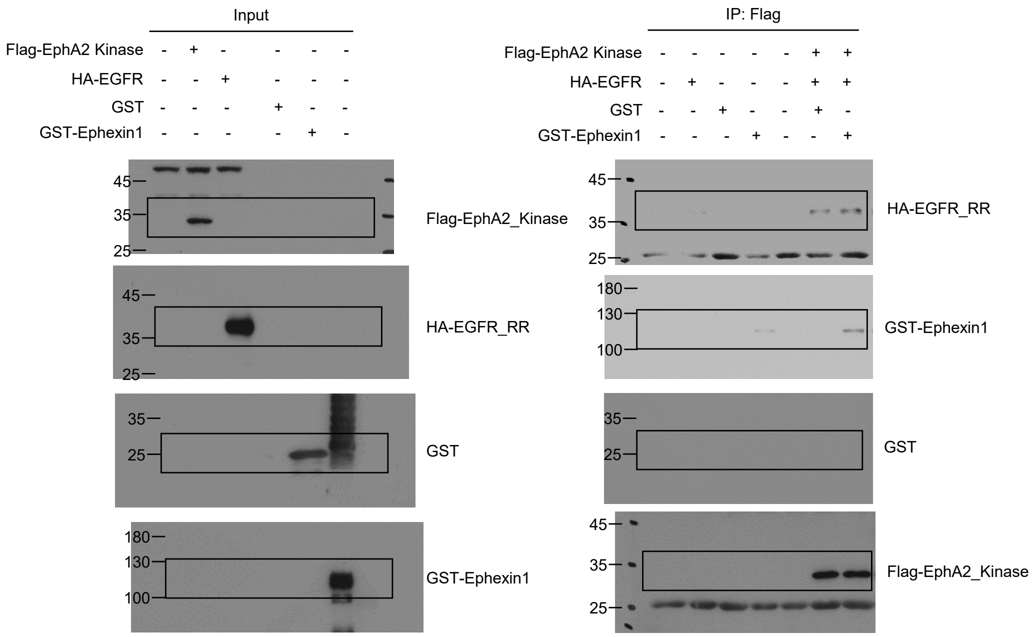

Figure 4a

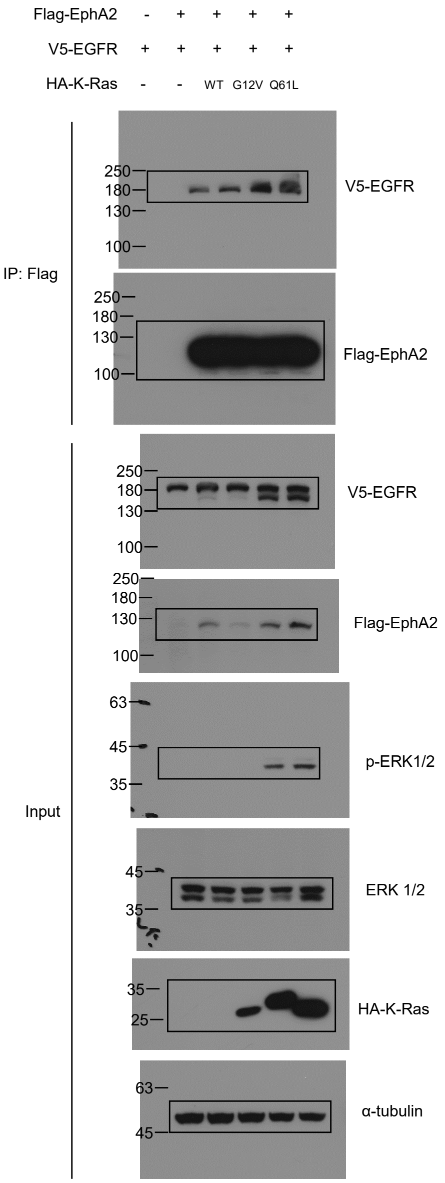

Figure 4b

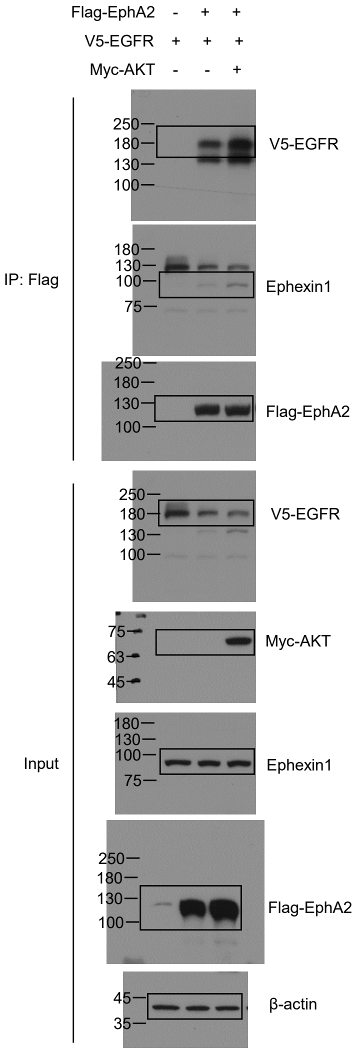

Figure 4c

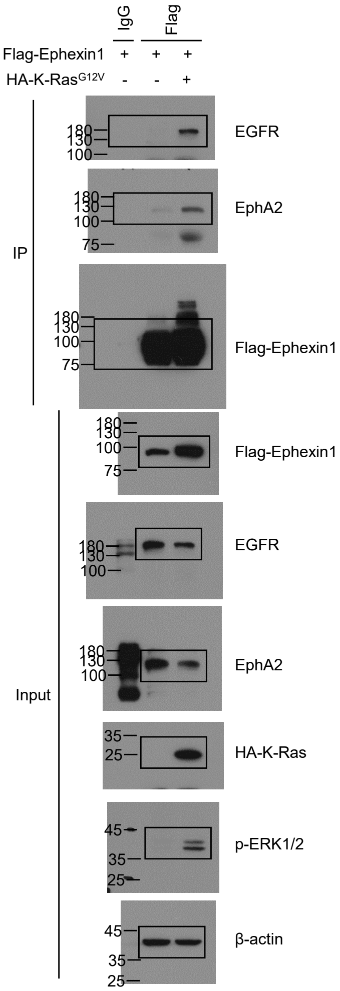

Figure 4d

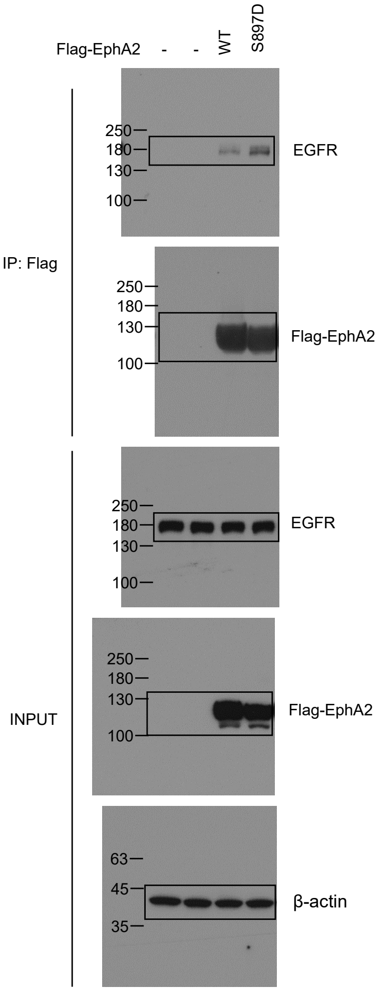

Figure 4e

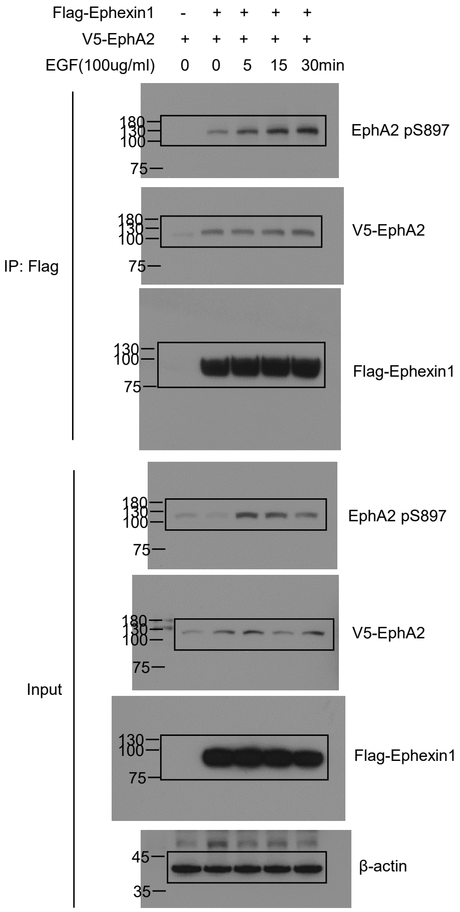

Figure 4f

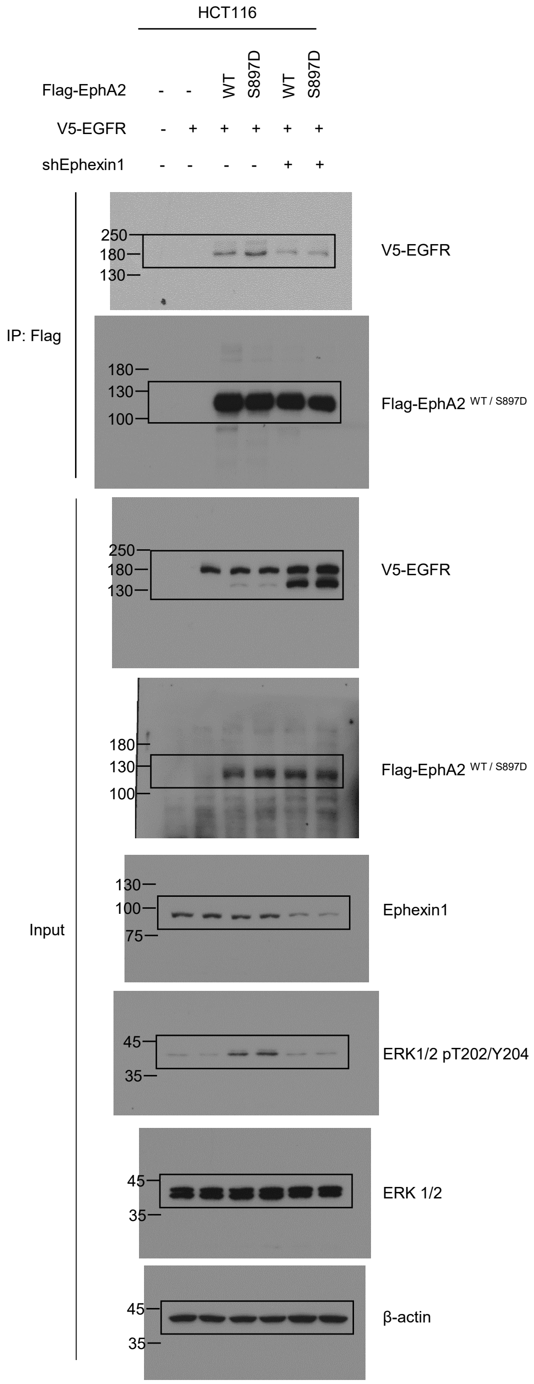

Figure 5a

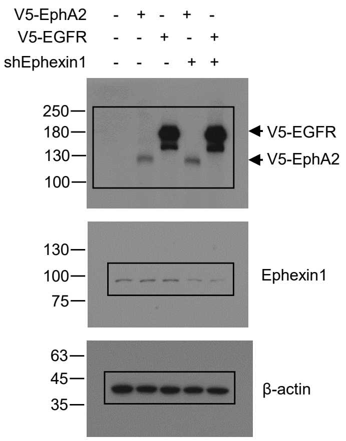

Figure 6a

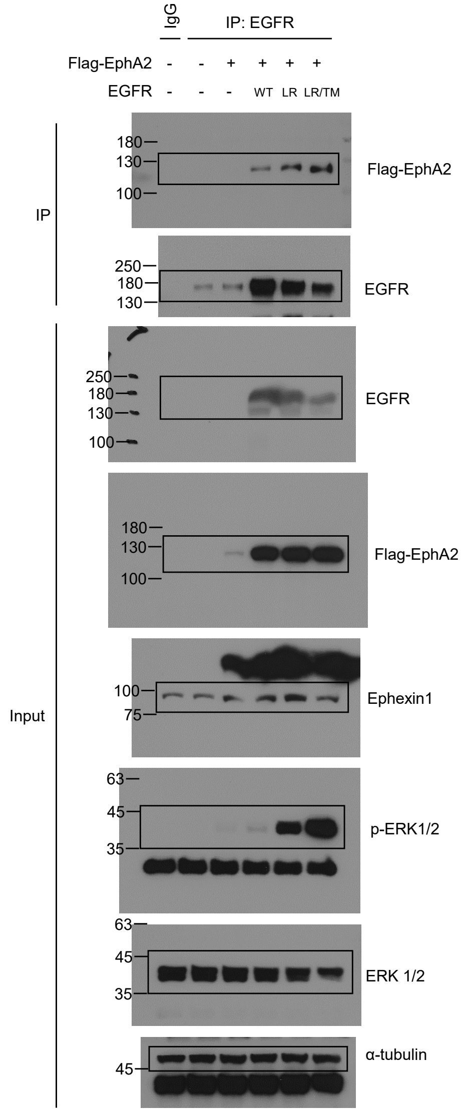

Figure 6b

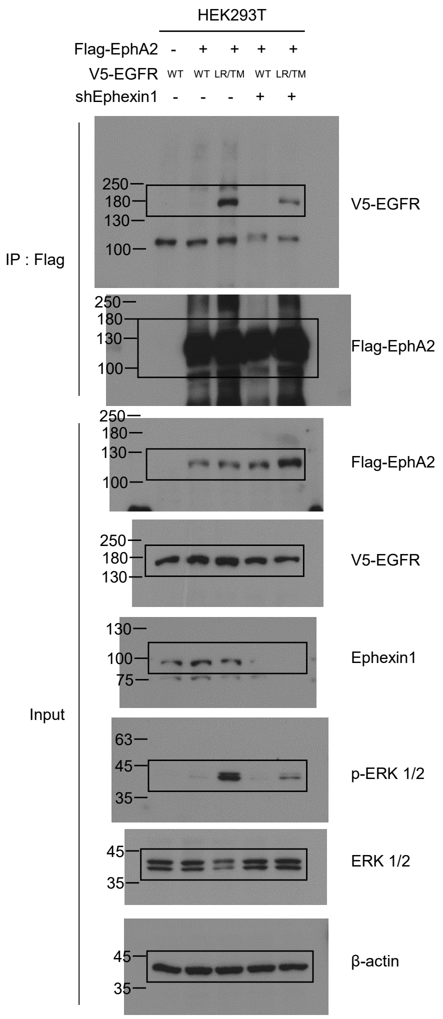

Figure 6c

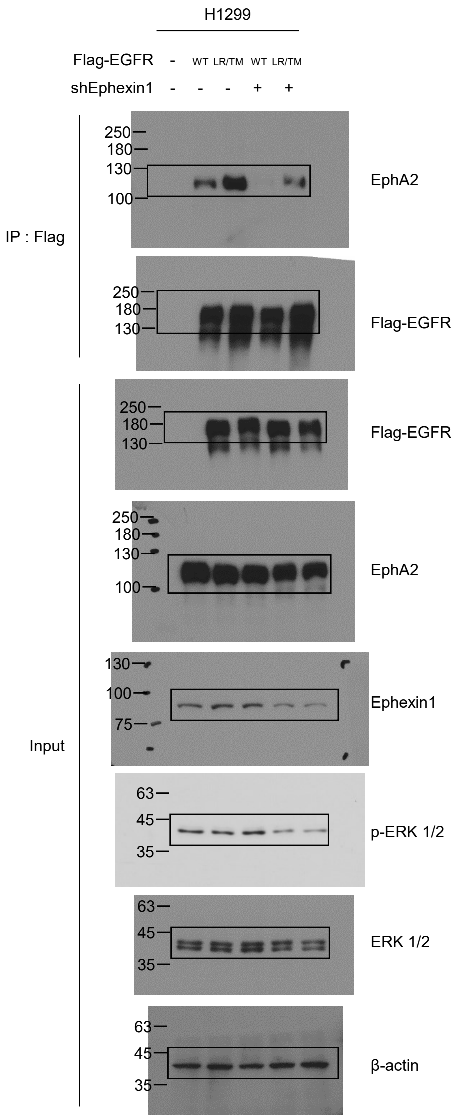

Figure S1. d

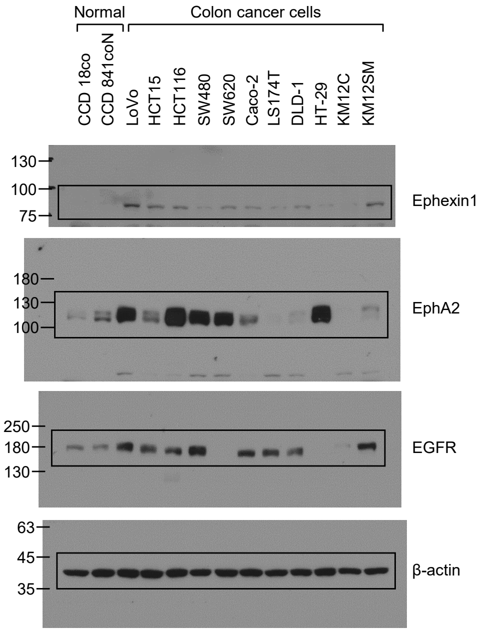

Figure S2. a

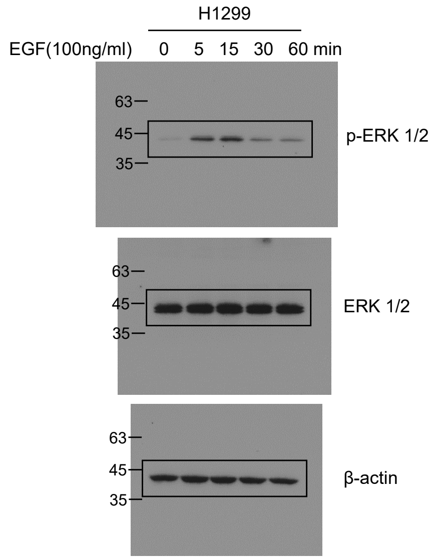

Figure S2. b

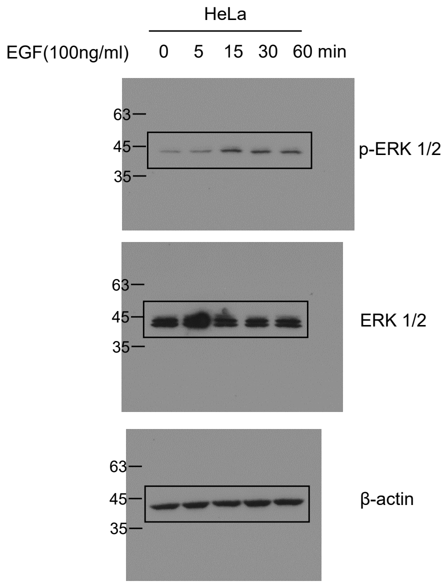

Figure S3. a

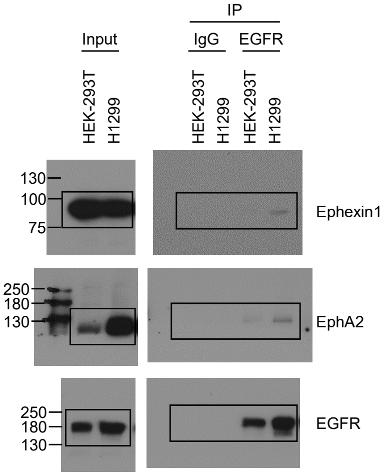

Figure S3. b

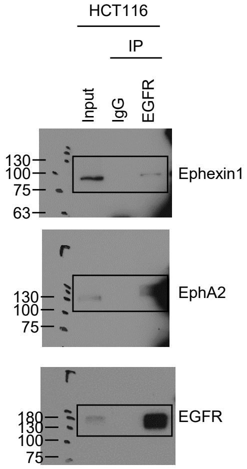

Figure S3. f

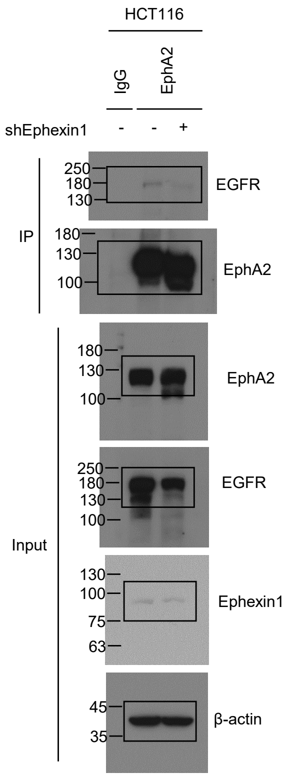

Supplement: Supplementary file 2 — Original Data File [file 41419_2022_4984_MOESM2_ESM.pdf]
